# Balanced Active Core in Heterogeneous Neuronal Networks

**DOI:** 10.3389/fncom.2018.00109

**Published:** 2019-01-29

**Authors:** Qing-long L. Gu, Songting Li, Wei P. Dai, Douglas Zhou, David Cai

**Affiliations:** ^1^School of Mathematical Sciences, MOE-LSC, and Institute of Natural Sciences, Shanghai Jiao Tong University, Shanghai, China; ^2^Department of Physics and Astronomy, and Institute of Natural Sciences, Shanghai Jiao Tong University, Shanghai, China; ^3^Courant Institute of Mathematical Sciences and Center for Neural Science, New York University, New York, NY, United States; ^4^NYUAD Institute, New York University Abu Dhabi, Abu Dhabi, United Arab Emirates

**Keywords:** balanced state, homogeneous, heterogeneous, active core, sparse coding, Fokker-Planck equation

## Abstract

It is hypothesized that cortical neuronal circuits operate in a global balanced state, i.e., the majority of neurons fire irregularly by receiving balanced inputs of excitation and inhibition. Meanwhile, it has been observed in experiments that sensory information is often sparsely encoded by only a small set of firing neurons, while neurons in the rest of the network are silent. The phenomenon of sparse coding challenges the hypothesis of a global balanced state in the brain. To reconcile this, here we address the issue of whether a balanced state can exist in a small number of firing neurons by taking account of the heterogeneity of network structure such as scale-free and small-world networks. We propose necessary conditions and show that, under these conditions, for sparsely but strongly connected heterogeneous networks with various types of single-neuron dynamics, despite the fact that the whole network receives external inputs, there is a small active subnetwork (active core) inherently embedded within it. The neurons in this active core have relatively high firing rates while the neurons in the rest of the network are quiescent. Surprisingly, although the whole network is heterogeneous and unbalanced, the active core possesses a balanced state and its connectivity structure is close to a homogeneous Erdös-Rényi network. The dynamics of the active core can be well-predicted using the Fokker-Planck equation. Our results suggest that the balanced state may be maintained by a small group of spiking neurons embedded in a large heterogeneous network in the brain. The existence of the small active core reconciles the balanced state and the sparse coding, and also provides a potential dynamical scenario underlying sparse coding in neuronal networks.

## 1. Introduction

Neuronal firing activity in the cortex can be highly irregular (Britten et al., 1993; Shadlen and Newsome, 1998; Compte et al., 2003; London et al., 2010). Because the precise timing of spikes may contain substantial information about the external stimuli, irregular activity may serve as a rich encoding and processing space for neural computation (Hertz and Prügel-Bennett, 1996; Gütig and Sompolinsky, 2006; Sussillo and Abbott, 2009; Monteforte and Wolf, 2012). To understand how the brain processes information, it is important to investigate how such irregularity emerges in the brain.

Some studies conclude that irregular firing may be regarded as noise, thus, conveying little information (Shadlen and Newsome, 1994; Han et al., 2015). Meanwhile, other studies show that timing of spikes and the temporal activity patterns of irregular neuronal firings *in vivo* are able to convey specific information (Richmond and Optican, 1990; Pillow et al., 2005; Whalley, 2013). A germinating mechanism underlying irregular activity was proposed in the balanced network theory (van Vreeswijk and Sompolinsky, 1996; Troyer and Miller, 1997; Vreeswijk and Sompolinsky, 1998; Vogels et al., 2005; Miura et al., 2007). In a balanced network, sparsely-connected neurons possess strong architectural coupling but weak pair-correlations in their activity. The excitatory and inhibitory inputs into each neuron, on average, dynamically balance, suppressing the mean of the total input. Consequently, fluctuations of the input become dynamically dominant, giving rise to irregular firing events of each neuron. The hallmarks of a balanced network include a broad and heterogeneous distribution of the single-neuron firing rate and a linear response of the mean population firing rate to the external input (Vreeswijk and Sompolinsky, 1998; Mehring et al., 2003; Renart et al., 2010). Consistent with theoretically predicted scenarios, certain experimental observations have been interpreted as consequences of balanced networks. For example, *in vitro*, the sustained irregular activity of neurons in slices of the ferret prefrontal and occipital cortex was shown to be driven by the balance of proportional excitation and inhibition (Shu et al., 2003). *In vivo*, the excitatory and inhibitory inputs to a neuron in ferret's prefrontal cortex were also found to be dynamically balanced (Haider et al., 2006).

As shown in recent experimental data, the structure of developing hippocampal networks in rats and mice conforms to a scale-free (SF) topology, with the number of connections per neuron following a power-law distribution (Bonifazi et al., 2009). Bidirectional and clustered three-neuron connection motifs were experimentally observed to occur with a frequency significantly above chance in the visual system (Song et al., 2005), thus strongly deviating from statistically homogeneous networks. The network in the somatosensory cortex of neonatal animals was found to be a small-world (SW) network (Perin et al., 2011), that is, its connectivity has properties of high clustering and short average path lengths (Newman, 2003b). These experimental observations show that the neuronal cortical connectivity is rather heterogeneous. Therefore, in this work, we investigate the influence of the wide distribution of the recurrent connectivity on neuronal network dynamics.

In general, it is theoretically challenging to understand the dynamical consequences of these complex network architectures (Boccaletti et al., 2006). Several studies have explored the dynamics of networks with heterogeneous connections. For instance, in Roxin (2011), the role of the broad degree distribution on the correlation of synaptic currents has been investigated. In addition, it has been observed that, in a heterogeneous network, a neuron with more presynaptic connections tends to fire less (Pyle and Rosenbaum, 2016). In a recent study (Landau et al., 2016), it has been found that a heterogeneous network is unbalanced in general because some neurons either never fire or fire fairly regularly in the network. The balanced state of the entire network (the global balanced state) can be achieved by setting strong correlations among the presynaptic excitatory, presynaptic inhibitory, and external inputs for each neuron, or through incorporating adaptation and plasticity into the dynamics of each neuron (Landau et al., 2016).

Theoretical and computational works so far have mainly focused on the global balanced state, i.e., each neuron in the network fires irregularly by receiving balanced excitation and inhibition. However, experimental studies have shown that information is often encoded by the firing of a relatively small set of neurons in the population, whereas other neurons in the network do not fire at all. This phenomenon is often referred to as sparse coding and has been observed in many cortical regions. For instance, sparse firing activity has been observed in the barrel cortex of mice (O'Connor et al., 2010), the auditory cortex of rats (Hromádka et al., 2008), and the primary olfactory cortex of rats (Poo and Isaacson, 2009), which is elicited by a variety of stimuli. Because a large proportion of neurons is silent during information processing, it is suggested that the global balanced state may not commonly exist in cortical regions.

Based on all the above observations, there are several important issues that remain to be clarified: whether the small group of those active neurons embedded in large heterogeneous neuronal networks can be in a balanced state and, if so, how such a balanced sate in the active subnetwork of heterogeneous networks differs from a global balanced state in homogeneous networks; whether the existence of a balanced active subnetwork sensitively depends on the topology of complex networks; what dynamical characteristics those active neurons have in order to maintain a balanced state in heterogeneous networks; how a balanced active subnetwork emerges from heterogeneous network dynamics; and what the dynamical implications of a balanced active subnetwork has for general complex networks. Below we will address these issues by investigating both the SF networks and SW networks with various types of single-node dynamics. Note that the definition of the SF network in our simulations deviates from the exact definition in which a network is called scale-free if its degree distribution exhibits power-law behavior, at least in its upper tail, i.e., *P*(*k*) ∝ *k*^−γ^ as *k* → +∞ (Reed, 2006). In numerical simulations, the power-law degree distribution we use takes the form as *P*(*k*) ∝ *k*^−γ^ for *k* ∈ [*K*_0_, *K*_1_]. Here, the degree of each neuron has a lower bound *K*_0_ determined as *K*_0_ ≈ 0.95*%N* according to an experimental observation (Bonifazi et al., 2009), where *N* is the network size. In addition, the degree of each neuron also has an upper bound *K*_1_ determined by Equations (8–10) to ensure that the network is sparsely connected. Because the mean connectivity of a sparse network is much smaller that the network size, the value of *K*_1_ is smaller than *N*.

## 2. Results

To contrast with networks of heterogeneous topologies below, we first recapitulate the balanced state in a homogeneous network, i.e., an Erd$\ddot{\text{o}}$s-Rényi (ER) network of binary neurons (Vreeswijk and Sompolinsky, 1998). In this balanced network, an important feature of its connectivity structure is that neurons are sparsely connected with strong synaptic strength. As discussed in section 4 specifically, the average number of connections *K* to each neuron from both presynaptic excitatory and presynaptic inhibitory populations is much smaller than the total number of neurons in the network, and the coupling strength is of the order $1/\sqrt{K}$. This scaling ensures persistent fluctuations of inputs in the large-*K* limit.

As shown in Figure S1, the hallmarks of the balanced state in a homogeneous neuronal network are summarized as follows: *balanced net input, irregular activity, stationary population-averaged activity, heterogeneity of firing rate, linear response*. A detailed description of the properties can be found in Figure S1 (Supplementary Material). All the balanced phenomena in the binary model can be demonstrated analytically from the standpoint of the classical balanced network theory (Vreeswijk and Sompolinsky, 1998). Note that both the theory and simulations are based on the assumptions that the network is homogeneous, i.e., of the ER type, and that the neuron is of the binary type. These assumptions are high simplifications of the biological reality. Biological neuronal networks tend not to be homogeneous, e.g., the connections can be of SF (Scannell et al., 1999; Sporns et al., 2004, 2007; Kaiser et al., 2007) or SW type (Sporns and Zwi, 2004; Sporns, 2006; Perin et al., 2011). In general, it is expected that the topology could strongly influence the dynamics of neuronal networks (Shkarayev et al., 2009). A natural and important extension of the theory is to examine the existence of a balanced state in heterogeneous networks. In the following, we first investigate the SF neuronal network, then discuss the case of the SW network. As an extension to the binary neuron model, we resort to the I&F model in our simulation (Carandini et al., 1996; Rauch et al., 2003; Cai et al., 2005; Rangan et al., 2005; Zhou et al., 2009, 2013).

### 2.1. Uncorrelated SF Network With I&F Neurons

In this section, we address the question of whether there exists balanced-network dynamics in an uncorrelated SF network using the current-based I&F neuronal model coupled with delta-pulse synaptic currents. This model is computationally simple but biologically more realistic than the binary model (the model details can be found in section 4).

Here, we focus on the SF topology with uncorrelated in-degree between neighboring neurons, and generate the SF networks with a given mean connectivity 2*K* (each neuron on average has *K* presynaptic excitatory neurons and *K* presynaptic inhibitory neurons). A network is called scale-free if its degree distribution exhibits power-law behavior, at least in its upper tail, i.e., *P*(*k*) ∝ *k*^−γ^ as *k* → +∞ (Reed, 2006). It should be pointed out that the mean connectivity 2*K* and the decay exponent γ of the power-law distribution are the two main factors that determine the SF network connectivity structure (details can be seen in section 4. We again invoke the coupling strength of order $1/\sqrt{K}$ to ensure that the network is fluctuation-driven when *K* is large. For each neuron in the network, the number of its presynaptic excitatory neurons is set to be highly correlated with that of presynaptic inhibitory neurons, consistent with the setting in the classic ER network as well as the experimental observation (Liu, 2004). The external input to each neuron is allowed to be uncorrelated with the cortical input, which will lead to the break of the global balanced state. However, it remains unclear whether a balanced state can exist in the subgroup consisting of the active neurons in such a network.

**Figure 1:**
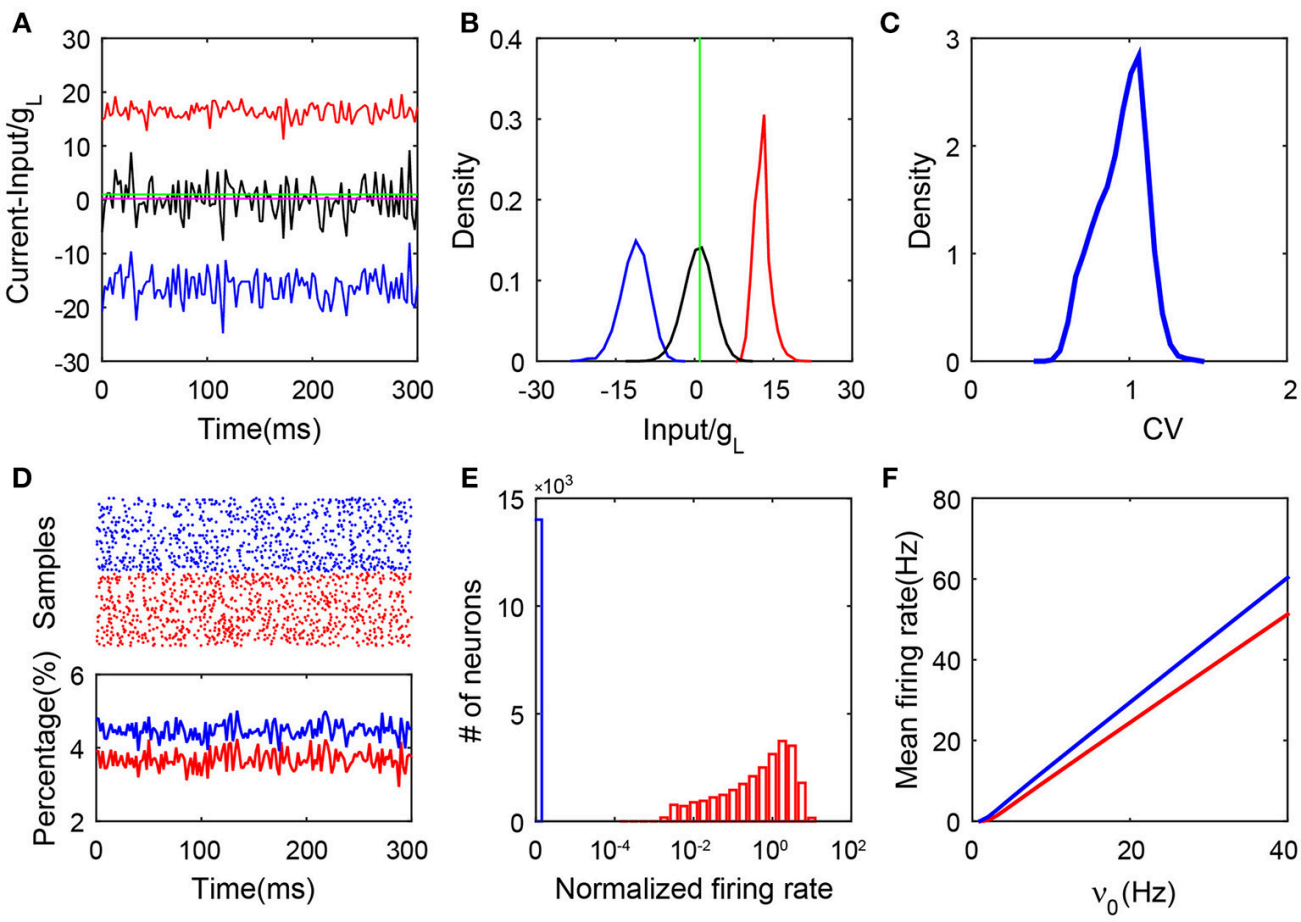
Properties of an SF balanced network with pulse-current-based I&F neurons. **(A)** The balanced excitatory and inhibitory inputs into a sample neuron (transient dynamics have been removed). The magnitudes of the excitatory (red) and inhibitory (blue) inputs (scaled by the leakage conductance *g*_*L*_) stay far away from the firing threshold (green), whereas the total input (black) (scaled by *g*_*L*_) crosses the threshold stochastically with its mean (magenta, the value is 0.29) remaining below the threshold; **(B)** The probability density functions of the excitatory (red), inhibitory (blue) and total (black) inputs (scaled by *g*_*L*_) for the sample neuron in **(A)**. The green line is the threshold; **(C)** The distribution of the CV value. Here, CV is calculated from the ISIs of each neuron; **(D)** The upper panel is the raster plot of a partial network (100 sample neurons selected at random from the network, with a time evolution of 300 ms), which exhibits asynchronous neuronal activity; the lower panel shows the percentage of the firing neurons over the network in each time window, where the time window is 2.5 ms. The transient dynamics have been removed; **(E)** The log-histogram of neuronal firing rates (normalized by the mean firing rate averaged across the entire network). The blue bar encodes quiescent neurons, and the red bars encode neurons with non-zero firing rates; **(F)** The mean firing rate of the excitatory (red) and inhibitory (blue) populations as a linear function of the external input. Here, $N_{E}=N_{I}=2\times 10^{4}$ and *K* = 400. In panels **(A–E)**, ν_0_ = 15 Hz. Other parameters are specified in section 4.

Our simulation results lead to the conclusion that only a group of neurons in this SF network can have firing activity and their dynamics follow a balanced state with all its hallmarks. In Figures 1A,B, we illustrate an example of the balance between the excitatory and inhibitory synaptic inputs to the firing neurons. We report the synaptic input at each moment by its time average within a small time window—we select a time bin of 2.5 ms. As shown in Figure S2, we can observe that the net input of each firing neuron can have a relatively small amplitude due to the cancellation of its excitatory and inhibitory parts. In addition, the firing rate of each individual active neuron is linearly correlated with its time-averaged net input, which is consistent with a recent study (Argaman and Golomb, 2018). Just as for neurons in the homogeneous balanced network, the CV value as shown in Figure 1C for the ISIs of each spiking neuron in the SF network is broadly distributed. This is consistent with the irregular activity of these neurons with heterogeneous connectivity. As shown in Figure 1D, the population activity is asynchronous and stationary as the percentage of firing neurons fluctuates in time around a constant with a small amplitude. In Figure 1E, we show that strong heterogeneity is captured by the bimodal distribution of the single-neuron firing rate. Compared with the distribution of firing rate in the homogeneous system (Figure S1E), the firing rate distribution in the SF case manifests a sharp peak near the origin (blue bar). Our result shows that there exists a group of neurons with no firing activity (we will further discuss the significance of this phenomenon below). We point out that a group of neurons with no firing activity has been previously found in other heterogeneous networks, e.g., the network with broad Gaussian distributions of degrees (Landau et al., 2016; Argaman and Golomb, 2018). Finally, in Figure 1F, we show the linear response of both the excitatory and inhibitory populations to the external rate. These features still exist asymptotically as the size of the network increases. In particular, the fluctuations of the synaptic currents received by active neurons do not vanish but are kept as order one, even for very large network size (Figure S3). To summarize, by the above hallmarks of the balanced state, the stationary state of those neurons with firing activity in the SF I&F neuronal network with delta-pulse synaptic currents can be readily identified as a balanced state.

### 2.2. Quiescent and Active Groups in the SF Network

As shown in Figure 1E, we find that the neuronal dynamics of SF networks separate the neuronal population into two subnetworks in our simulations: one consisting of neurons that fire no spikes (blue bar), which will be referred to as the quiescent group; the other consisting of firing neurons (red bar), referred to as the active group (core). Subsequently, we investigate the mechanism underlying how the SF network system evolves into these two different groups and what are the characteristics of dynamics for neurons in these two groups.

**Figure 2:**
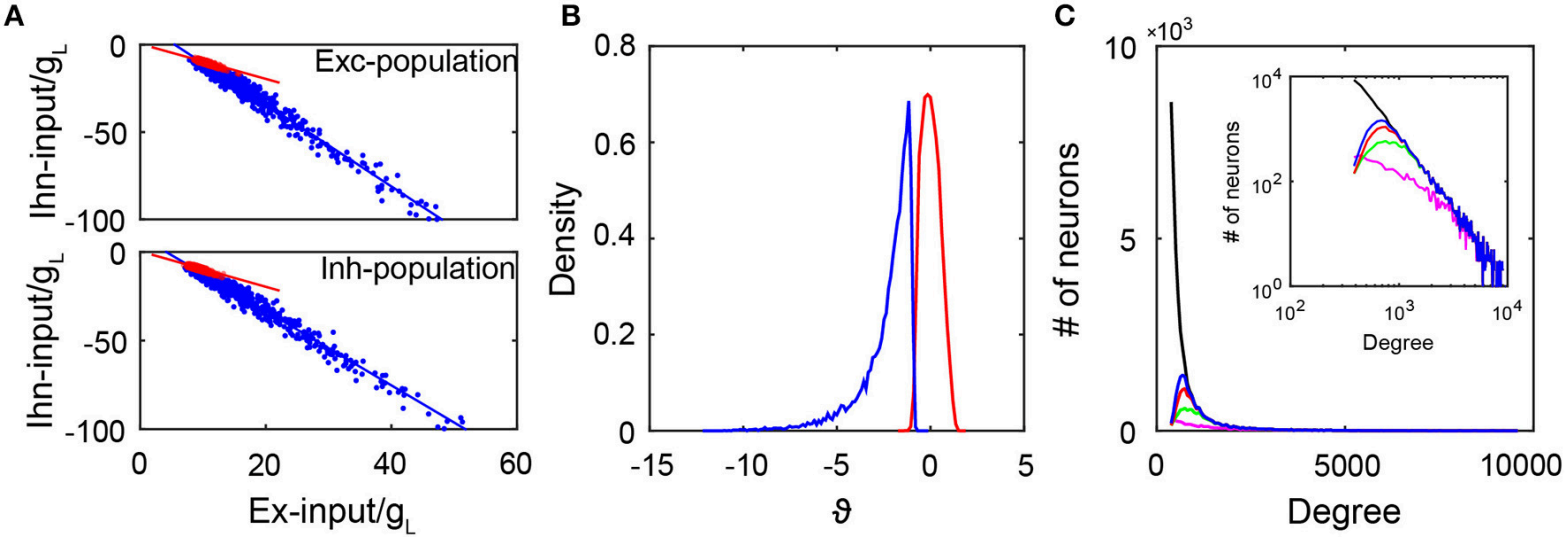
The subgroups in the SF network. **(A)** The excitatory and inhibitory inputs (normalized by *g*_*L*_) into active neurons (red dots) and quiescent neurons (blue dots). The upper panel is for the excitatory population with slopes of −1 (red line) and −2.35 (blue line). The lower panel is for the inhibitory population with slopes of −1 (red line) and −2.11 (blue line). Red and blue lines are linear fitting of the red and blue dots respectively. Here, we select 1,000 active and 1,000 inactive neurons randomly for the plot; **(B)** The distribution of ϑ as the time average of the total input into each neuron normalized by its standard deviation. Blue line is for the quiescent subgroup, and red line is for the active subgroup; **(C)** The degree distributions of the entire network (black solid line) and that of neurons in the quiescent group for different coupling strength ratio ϕ = *J*_*EI*_/*J*_*EE*_. The insert is the log-log plot for the same distributions. In our simulations, we fix *J*_*II*_/*J*_*EI*_ = 0.9. Here, ϕ = 3 for the blue solid line, ϕ = 2 for the red solid line, ϕ = 1.5 for the green solid line, and ϕ = 1.2 for the magenta solid line. The distributions agree with one another in the region of large degrees. Data in **(A,B)** are from the case in Figure 1.

In a balanced network, the excitatory and inhibitory inputs to each neuron need to approximately cancel each other. Therefore, the mean-field balanced conditions in the large-*K* limit shall hold (Vreeswijk and Sompolinsky, 1998) as follows:

(1) $$\begin{array}{r} \begin{array}{c} KJ_{EE}m_{E}+f_{E}\nu_{E}=-KJ_{EI}m_{I},\\ KJ_{IE}m_{E}+f_{I}\nu_{I}=-KJ_{II}m_{I}, \end{array} \end{array}$$

where *m*_α_ is the mean firing rate of the αth population, *J*_αβ_ is the coupling strength from the βth population to the αth population, and *f*_α_ and ν_α_ are the strength and the rate of the external Poisson input to the αth population for α, β = *E, I*. As shown in Figure 2A, the excitatory and inhibtory inputs are indeed proportional to each other in both the active and quiescent groups. This is consistent with a recent experimental observation (Xue et al., 2014). In addition, it can be clearly observed that the quiescent group is strongly inhibited because the inhibitory input in the quiescent group is more than twice that in the active group given the same excitatory input. By calculating the time-averaged total input to a neuron normalized by its standard deviation and denoting as ϑ, it is clear from Figure 2B that the distribution of ϑ has a long negative tail for the quiescent group. Consequently, rarely can fluctuations drive their membrane potentials across the threshold. Note that the distribution of ϑ is concentrated around zero for the active group, thus indicating the neurons in the active group have fluctuation-dominated inputs. In addition, the fact that the quiescent group is strongly inhibited can also be reflected in the cross-correlation structure between the excitatory and inhibitory synaptic inputs to each neuron in the quiescent group (Roxin, 2011). As shown in Figure S4A, the average cross-correlation is higher for neurons in the quiescent group than that in the active group, indicating that the increase of the excitatory input is quickly followed and canceled by the inhibitory input such that a neuron in the quiescent group is more inhibited than a neuron in the active group at each moment. Therefore, neurons in the quiescent group are not in the balanced state.

Next, we investigate the issue of how the coupling strength of the network affects the emergence of the active group. In particular, we focus on the competition between the excitatory and inhibitory coupling strength quantified by the ratio ϕ = *J*_*EI*_/*J*_*EE*_ with fixed *J*_*II*_/*J*_*EI*_. In our simulation, we fix the network topology while varying the value of ϕ. Note that the degree distribution of the entire network is given by an SF network construction, thus independent of ϕ; while the degree distribution of the active group depends on ϕ — different coupling strengths give rise to different dynamics, which in turn generate different active subnetworks dynamically. Figure 2C displays the degree distribution of the entire network and those of the quiescent groups with different values of ϕ. It is important to observe that these degree distributions agree with one another in the region of large degrees, that is, the quiescent group tends to be composed of the neurons with a large degree. This is consistent with a recent observation (Pyle and Rosenbaum, 2016) that a neuron with a larger degree tends to fire less. Because the neurons in the quiescent group have large degrees, each pair of them tend to share a large amount of common inputs. As shown in Figures S4B,C, by calculating the cross-correlation between the excitatory inputs or inhibitory inputs received by two neurons from the same group, we find that the average cross-correlation value between two inputs of the same type (excitatory or inhibitory) across all pairs of neurons in the quiescent group is indeed significantly greater than that in the active group.

Next, we deploy the coarse-grained approach to further deepen our understanding of the dynamics in this SF system.

### 2.3. Fokker-Planck Analysis of the SF Network Dynamics

From the mean-field balanced conditions (1), one can obtain the relationship between the population-averaged mean firing rate and the external drive:

(2) $$\begin{array}{r} m_{E}=\frac{1}{K}\frac{J_{II}f_{E}\nu_{E}-J_{EI}f_{I}\nu_{I}}{J_{EI}J_{IE}-J_{II}J_{EE}},\;m_{I}=\frac{1}{K}\frac{J_{IE}f_{E}\nu_{E}-J_{EE}f_{I}\nu_{I}}{J_{EI}J_{IE}-J_{II}J_{EE}}. \end{array}$$

**Figure 3:**
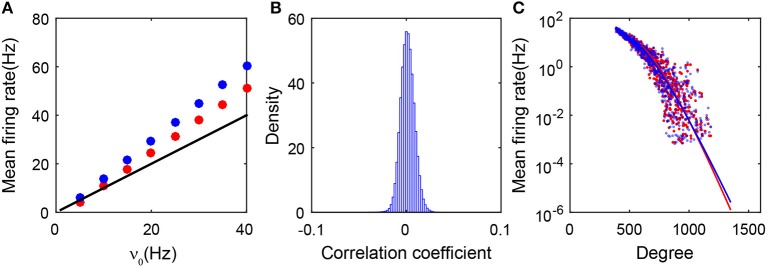
Theoretical analysis of the SF network. **(A)** Gain curves. The black solid line is obtained from the balanced condition. The theoretical gain curves for the excitatory and inhibitory populations overlap. The red dots (excitatory population) and blue dots (inhibitory population) are obtained from the simulation; **(B)** The distribution of the cross-correlation coefficient between spike trains of all pairs of neurons in the entire network. It is narrowly centered around zero; **(C)** The mean firing rate of each neuron ensemble as a function of its degree. Red dots and blue dots are from the simulation. The red and blue lines are obtained from the FP approximation by Equation (22). Red and blue colors encode excitatory and inhibitory populations, respectively. Data in panels **(A–C)** are from the case in Figure 1.

As shown in Figure 3A, the predictions of the balanced conditions Equation (2) cannot adequately capture the linear response of the population-averaged mean firing rates to the external inputs obtained in the simulation. To understand quantitatively the influence of the degree heterogeneity of the SF network, we perform the analysis of the Fokker-Planck (FP) equations corresponding to the network dynamics below.

As shown in Figure 3B, we first note that the firing events between neurons are extremely weakly correlated in the SF network. Therefore, the input into each neuron in the system can be regarded as three Poisson trains (Cinlar, 1972): the external, the excitatory, and the inhibitory synaptic inputs. Accordingly, we can derive the FP equation to describe the dynamics of an I&F neuron with Poisson inputs (Brunel, 2000; Cai et al., 2006). To derive the FP equation for a group of coupled neurons, we need to take the structure of the SF network into account (see section 4 for details). By treating all the neurons that possess the same number of presynaptic neurons as one ensemble, we then derive the FP equation for each ensemble and further obtain its stationary-state solution. We find that the mean firing rate *m*^*k*^ for the *k*th ensemble decays exponentially with the neuronal degree *k* in that ensemble. Consequently, neurons with a sufficiently large degree will not fire or have extremely low firing rates that can barely be detected in numerical results with a finite simulation time, thus they will be classified into the quiescent group. The exponential decay of the firing rate from the FP analysis has been further verified in numerical simulations as shown in Figure 3C.

### 2.4. Balanced Active Core and Conditions for Its Existence

From the above discussion, it can be clearly seen that the entire SF network is unbalanced, whereas the active subnetwork is balanced. We now focus on the balanced subnetwork that contains only the active neurons and the connectivity structure of these neurons. We will refer to this subnetwork as an active core, which captures the spiking activity and the effective communication of the entire neuronal network.

**Figure 4:**
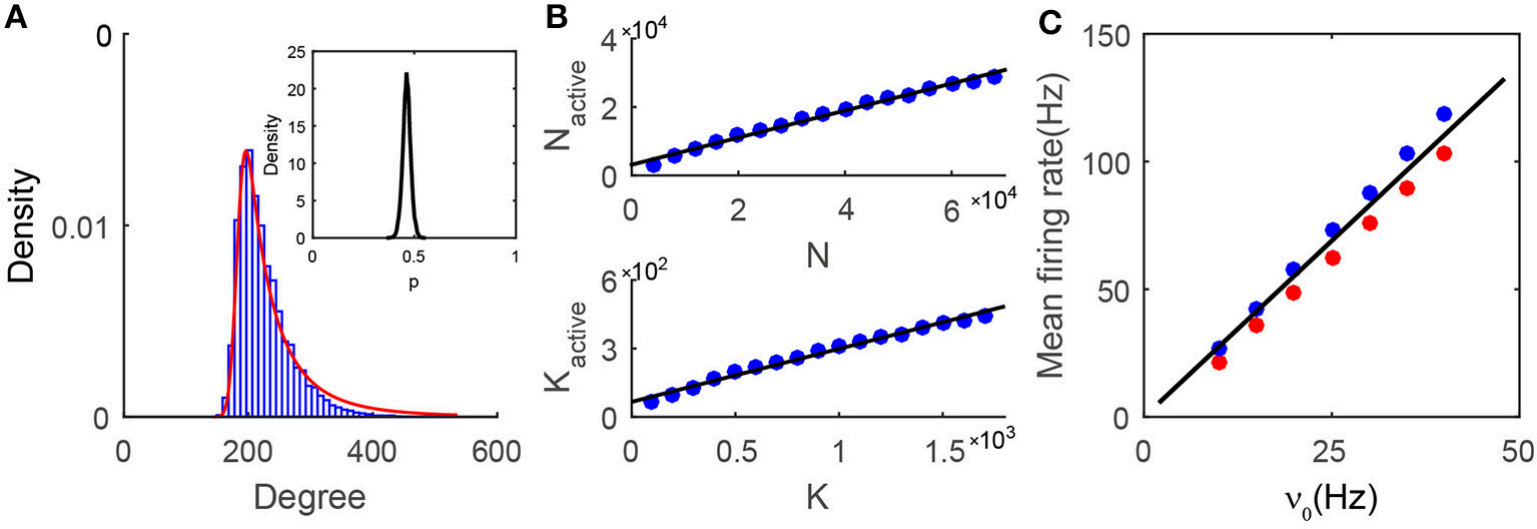
Properties of the active core. **(A)** The degree distribution in the active core. Numerical results (blue bars) can be well fitted by our prediction (Equation 3, red line). The insert is the distribution of *p* from the numerical simulation. For any single neuron, *p* is the fraction of the number of its active presynaptic neurons over the number of its total presynaptic neurons. The distribution is narrowly centered around a constant; **(B)** Relationship between the active core size and the network size. In the simulations, the sparsity *K*/*N* = 0.025 is fixed, while *N* and *K* vary in different cases. In each network of different size, we choose *K*_0_ ≈ 0.95*%N*, and the value of *K*_1_ according to Equation (8). The size (upper) and the mean connectivity (lower) of the active core both grow linearly with those of the entire network. The black solid line is a linear fitting of the simulation results (blue dots), with *R*^2^ = 0.993 for the upper panel and *R*^2^ = 0.990 for the lower panel; **(C)** The linear population response to the external drive in the active core. Black solid line is the prediction from the mean-field balanced conditions in the active core. Red (excitatory population) and blue (inhibitory population) dots are obtained from the simulation results. Data in **(A,C)** is from the case shown in Figure 1.

We first investigate the issue of how to quantitatively characterize the features of the active core. From Figure 4A, it is important to note that the degree distribution of the neurons in the active core is sharply peaked, resembling that of neurons in homogeneous networks. Why does the degree distribution of the active core in the heterogeneous SF network possess the characteristics of a homogeneous ER network? For each neuron, we first examine the fraction of its active presynaptic neurons amongst all its presynaptic neurons, which will be denoted as *p* below. The distribution of *p* as shown in the insert of Figure 4A is sufficiently narrow to be approximated as a constant. In general, the value *p* will be affected by the E-I input strength ratio ϕ and the decay exponent of the degree distribution γ, as shown in Figure S5. Note that, for each neuron, *p* can also be viewed as the probability of finding one of its presynaptic neurons to be active. The probability of finding a neuron with *w* active presynaptic neurons can then be derived from the law of total probability, $P(w)=\sum_{k}P(k)P(w|k)$, where *P*(*k*) is the probability of finding a neuron having *k* presynaptic neurons, as the case here, whose distribution follows a power-law, *P*(*k*) = *ck*^−γ^. By ignoring the correlation between the degree distribution of the active core and the formation of the active core, the conditional probability *P*(*w*|*k*) can be approximated by a binomial distribution $P\left(w|k\right)=C_{k}^{w}p^{w}{\left(1-p\right)}^{k-w}$. Further approximating the binomial distribution by a Gaussian, we can derive an approximation for *P*(*w*):

(3) $$\begin{array}{c} P(w)\approx \sum_{k}ck^{-\gamma }\frac{1}{\sqrt{2\pi kp(1-p)}}\exp\left[-\frac{{\left(w-pk\right)}^{2}}{2kp(1-p)}\right]. \end{array}$$

The probability *P*(*w*) is a sum of a series of Gaussian terms with the coefficient of each term weighted by *k*^−γ^. Therefore, a larger value of *k* has a smaller contribution to the sum. In particular, for sufficiently large γ, the dominant term can exactly be a Gaussian. When γ is *O*(1) as set in our simulations, the degree distribution of the active core still resembles a Gaussian and can be captured by Equation (3). As shown in Figure 4A and Figures S5B–D, the prediction by Equation (3) is in very good agreement with the measured degree distribution of the active core for various values of γ.

Denoting the size and mean connectivity (in-degree) in the active core as *N*_active_ and *K*_active_ respectively, we next examine the relationship between *N*_active_ and *N* as well as *K*_active_ and *K*. Recall that *K* is the average presynaptic connectivity of the original SF network. Numerically, as shown in Figure 4B, *N*_active_ and *K*_active_ increase linearly with *N* and *K* respectively. As a consequence, when *K* → +∞, *N* → +∞, we also have *K*_active_ → +∞, *N*_active_ → +∞. Therefore, the dynamics of the active core possess the same asymptotic behaviors as those of an ER network in the large-K limit.

By considering the active core as a homogeneous network, we can numerically solve its population-averaged mean firing rate from the following equations derived from the balanced condition in the large-*K* (*K*_active_) limit

(4) $$\begin{array}{l} m_{\text{active},E}=\frac{1}{K_{\text{active}}}\frac{J_{II}f_{E}\nu_{E}-J_{EI}f_{I}\nu_{I}}{J_{EI}J_{IE}-J_{II}J_{EE}},\\ m_{\text{active},I}=\frac{1}{K_{\text{active}}}\frac{J_{IE}f_{E}\nu_{E}-J_{EE}f_{I}\nu_{I}}{J_{EI}J_{IE}-J_{II}J_{EE}}, \end{array}$$

where the averaged connectivity of the active core *K*_active_ is read out from the simulation. As shown in Figure 4C, the linear response property of the active core can be well-captured by the predictions from Equation (4). The successful prediction also suggests the validity of the assumption in the analysis that the active core can be viewed as a balanced homogeneous network.

Note that the active core encompasses all spike events in the SF neuronal network, with connectivity similar to that of an ER network. The characteristics of the balanced state persist in the active core, that is the properties of *balanced net input, irregular activity, stationary population-averaged activity, heterogeneity of firing rate*, and *linear response* all hold. Clearly, our results demonstrate that there exists a balanced active core in the SF neuronal network.

Next, through theoretical analysis and numerical simulations, we have found that, in order to obtain the balanced active core, the following three conditions shall hold:

1. The cortical and external input strengths shall satisfy the following relation
  (5) $$\begin{array}{r} \frac{f_{E}\nu_{E}}{f_{I}\nu_{I}}>\frac{J_{EI}}{J_{II}}>1, \end{array}$$
  which can be derived from the balanced condition (Equation 4) by requiring the firing rates to be positive values and setting *J*_*IE*_ = *J*_*EE*_ for simplicity. Note that Equation (5) is consistent with the conditions derived from a homogeneous network (Vreeswijk and Sompolinsky, 1998).
2. The excitatory and inhibitory in-degrees for each neuron shall be highly correlated. This condition is consistent with the experimental observation that a conserved ratio of the numbers of excitatory and inhibitory synapses has been observed throughout the dendrites of cultured hippocampal neurons (Liu, 2004).
3. The smallest degree *K*_0_ in the network is required to be the same order as the population-averaged degree *K*. In fact, by analyzing the FP equation of the neuronal ensemble with the smallest degree *K*_0_, the total input to each neuron in this ensemble shall be close to zero in order to achieve the balance between the excitatory and inhibitory inputs, i.e.,
  (6) $$\begin{array}{r} fK\nu_{0}+J_{\alpha E}\frac{\Gamma }{1+\Gamma }r_{E}K_{0}-J_{\alpha I}\frac{1}{1+\Gamma }r_{I}K_{0}\approx 0, \end{array}$$
  where *f* is the external input strength, ν_0_, *r*_*E*_, and *r*_*I*_ are the average firing rates of the presynaptic external, cortical excitatory, and cortical inhibitory neuronal populations, respectively, *K* is the number of the presynaptic external neurons identical to the mean connectivity of the SF network, Γ is the ratio between the number of each neuron's presynaptic excitatory neurons to the number of each neuron's presynaptic inhibitory neurons. Because *f*, *J*_α*E*_, and *J*_α*I*_ are of order $O\left(1/\sqrt{K}\right)$, ν_0_, *r*_*E*_, *r*_*I*_, and Γ are of order O(1), *K*_0_ is required to be the same order as *K* in order to make the total input canceled out.

**Figure 5:**
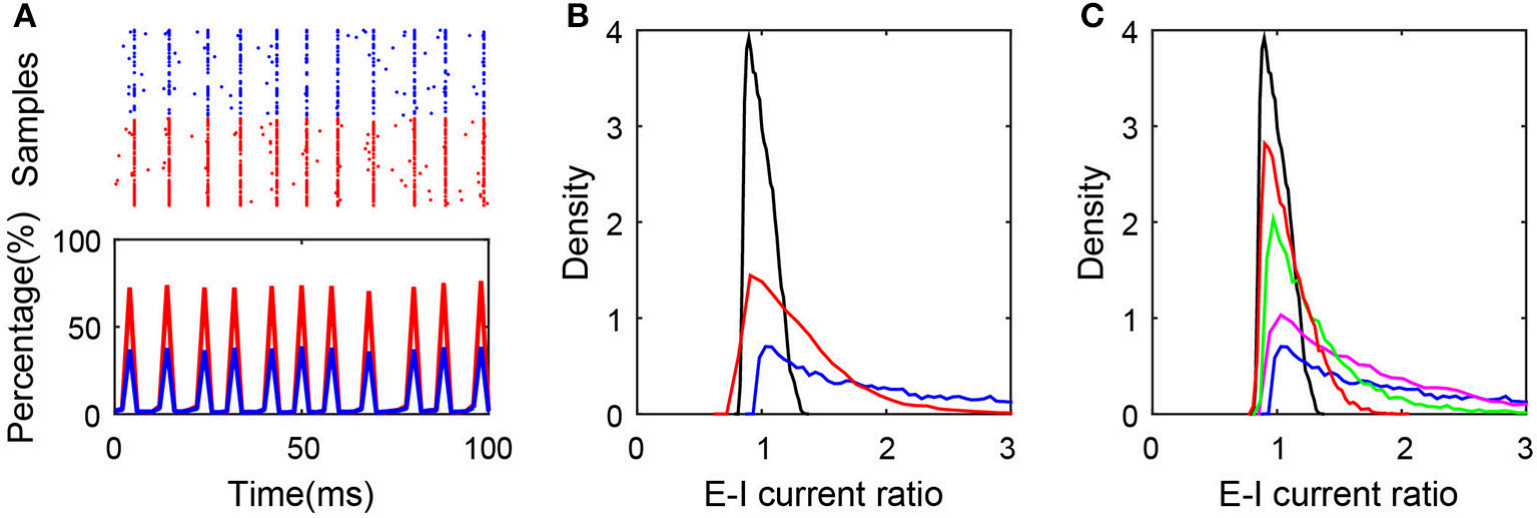
Conditions for the existence of the balanced active core. **(A)** Synchronized network dynamics induced by the break of balanced condition (1). In the simulation, $J_{EI}=1.8/\sqrt{K},J_{II}=2.0/\sqrt{K},f_{E}\nu_{E}=15\sqrt{K}$ and $f_{I}\nu_{I}=12\sqrt{K}$, where *K* = 400. The raster plot (upper panel) and the percentage of firing neurons over time indicate the synchronous dynamics in the system. Red and blue color encode the excitatory and inhibitory population, respectively; **(B)** The distributions of E-I current ratio in SF networks satisfying all the three conditions (black), after breaking condition (2) (blue), and after breaking condition (3) (red). The blue curve is plotted by setting the cross-correlation coefficient of the presynaptic excitatory degree and inhibitory degree as 0.2; and the red curve is plotted by setting *K*_0_ = 80, *K* = 400; **(C)** The ratio distribution corresponding to the SF network with different cross-correlation coefficients of the presynaptic excitatory degree and inhibitory degree. The cross-correlation coefficient equals 0.99 (black), 0.8 (red), 0.6 (green), 0.4 (magenta), and 0.2 (blue).

As one example shown in Figure 5A, breaking the first condition results in the synchronous state of the network. In addition, as shown in Figure 5B, breaking the second or third condition results in the deviation of the ratio from excitatory to inhibitory current input to neurons in the active group from unity, which obviously breaks the balanced input condition and the balanced state of the subnetwork as a consequence. In order to keep the excitation and inhibition balanced in the active neurons, it requires sufficiently high correlation between the numbers of excitatory and inhibitory presynaptic connections as shown in Figure 5C. Note that these conditions for the existence of the balanced active core are different from that proposed by the previous works for the existence of the global balanced state (Landau et al., 2016), in which the number of cortical inhibitory input is required to be correlated with both the number of cortical excitatory inputs and the number of external inputs. In contrast, the existence of a balanced active core only requires that the number of cortical inhibitory inputs be correlated with that of cortical excitatory input but not that of external input.

### 2.5. Correlated SF Networks With I&F Neurons

Because the architectural degree-correlation may play an important role in the dynamics of a system (Shkarayev et al., 2009), we generate SF networks with degree-correlation between neighboring nodes using a reshuffling strategy (Xulvi-Brunet and Sokolov, 2005). A balanced active core can still arise in correlated SF neuronal networks. An example is shown in Figure S6, in which the five hallmarks of *balanced net input, irregular activity, stationary population-averaged activity, heterogeneity of firing rate*, and *linear response* again perseverate robustly. The degree distribution exponent γ of the SF network used here is γ = 2.6, which is the same as that of the SF network for the uncorrelated case reported above (Figure 1). The degree correlation coefficient for the SF network in Figure S6 is ρ = 0.03. Similar to an SF network without degree correlation, the distribution of single neuron firing rates also possesses a high peak at zero. The SF network with degree correlation can also be decomposed into two subnetworks of distinct dynamics characterized by their firing rates. Figure S7B demonstrates that the structure of the corresponding active core also displays that of homogeneous networks.

By generating SF networks with different correlation coefficients and with γ = 2.6, all these SF systems exhibit dynamics with a balanced active core. The degree distribution of the active core can be successfully described by Equation (3) for all values of ρ ranging from −0.3 to 0.31 as shown in Figure S7. The properties of the dynamics in these active cores are again similar to those of an ER balanced network.

In summary, our results show that the degree correlation between different nodes does not affect the properties of the balanced active core in SF networks. For SF neuronal networks with degree correlations, the existence of the active core persists with the structure similar to that of an ER network and the active core possesses all the characteristics of the balanced state.

## 3. Discussion

In this work, we have shown that a sparsely but strongly connected SF network of I&F neurons can reach the balanced state in the active subgroup if the network satisfies three conditions: the inequality of the ratio of external and coupling strength between excitation and inhibition, high correlation between the numbers of presynaptic cortical excitatory and presynaptic cortical inhibitory neurons for each neuron, and the order of the smallest degree *K*_0_ being the same as that of the population averaged degree *K*. Despite the fact that all neurons in the SF network receive external inputs, the network is naturally separated into two subnetworks: one is the quiescent group consisting of silent neurons and the other is the active group consisting of neurons with non-zero firing rates. The separation of active and quiescent subgroups has also been observed in other heterogeneous networks with broad Gaussian degree distributions (Landau et al., 2016; Argaman and Golomb, 2018). The subnetwork consisting of all the active neurons with the connections between these neurons is then defined as the *active core* here. From our simulation, this active core possesses a degree-distribution characteristic of a homogeneous ER network, which can be described well by our theoretical analysis (Equation 3). In addition, the active core displays similar dynamical properties of the balanced state of an ER network (Figures 1, 4).

In addition, our results suggest that the balanced active core can be found in various heterogeneous networks as the results shown below. We also find that the silent neurons always possess larger degrees than the active neurons. This can be understood intuitively as follows: if the number of in-degrees in the external input is fixed, and the ratio of the numbers of excitatory and inhibitory synapses is maintained, neurons in the heterogeneous network that receive a large number of recurrent connections receive effectively more inhibition and are therefore silent.

### 3.1. Balanced Active Core in Other Networks

In addition to the pulse-coupled I&F neurons, for the SF network of either binary neurons or smooth-current-based I&F neurons, as shown in Figures S8–S10, the balanced active core also can be found. These results imply that the existence of the balanced active core is robust with respect to detailed single-neuron dynamics.

It has been shown that different architectural degree-correlations can induce different dynamical properties in SF networks (Krapivsky and Redner, 2001). These correlations can strongly influence the dynamics of the system (Shkarayev et al., 2009). However, as far as the balanced state is concerned, there still exists a balanced active core in SF neuronal networks with degree correlations as shown in Figures S6, S7, in which an ER-like active core controls its dynamics. In addition, the properties of the balanced state have been studied for various SF networks with a different decay exponent of degree distribution γ. As shown in Figures S5, S11–S13, the value of γ does not affect the existence of the balanced active core, but affects the size of the active core.

As is shown that certain neuronal networks in the brain exhibit small-world (SW) characteristics (Perin et al., 2011), we have also conducted simulations with SW connectivity. An active core of the balanced dynamics is again observed in the SW neuronal network with different rewiring probabilities (Figure S14). The degree distribution in the active core is still close to that of an ER network. Our results suggest that the balanced state embedded in the active core may broadly exist for various heterogeneous networks (Figure 4 and Figures S5, S7, S10, S14).

### 3.2. Heterogeneity in External Input

Accounting for the fact that the external inputs may vary from neuron to neuron, we have also examined the case of heterogeneous inputs in the simulation. Here, we choose the rate of the external input to the *i*th neuron in the αth population $\nu_{\alpha }^{i}$ from a Gaussian probability distribution with its mean ν_α_ and standard deviation CV·ν_α_ for α = E, I, where CV is the coefficient of variation. As shown in Figure S15, for CV ranging from 0.1 to 0.4, we can still observe the existence of the active core, in which neurons receive balanced excitatory and inhibitory inputs. This indicates that the the broadly-distributed external input may not affect the existence of the active core (Figure S16).

In addition, Figures S17A,B provides an example of the heterogeneous strength of the external input following a log-normal distribution (Song et al., 2005) with a uniformly-distributed rate for different neurons. For this case, the dynamics still manifest a balanced active core whose in-degree distribution is again in excellent agreement with the prediction of Equation (3) shown in Figure S17C. These results may suggest that the active core can exist for various external inputs.

### 3.3. Biological Relevance of the Balanced Active Core

Many neuronal networks in the brain exhibit statistically heterogeneous connectivity structures. It has been observed that the connections of the neurons in layer 5 of the rat visual cortex display various highly clustered three-neuron connectivity patterns (Song et al., 2005). In addition, neuronal connectivity has been found to possess SF properties in rat hippocampal networks (Bonifazi et al., 2009). The network connectivity between neurons in the somatosensory cortex of neonatal animals possesses the attributes of a SW network (Perin et al., 2011).

In addition, experimental studies have shown that there often exists a small subnetwork of highly active neurons along with a large proportion of neurons being silent in neocortex of the brain. For example, during a head-fixed object localization task, only about half of all the neurons in a barrel column have been found to fire (O'Connor et al., 2010). Experimental recordings in the primary auditory cortex of unanesthetized rats have shown that 50% of the neural population failed to respond to any of the simple stimuli (Hromádka et al., 2008). Furthermore, *in vivo*, each odor can only evoke the activity of about 10% of neurons from anterior piriform cortex Layer 2/3 (Poo and Isaacson, 2009).

Our results show that, starting from heterogeneous network connectivity, the emergent network dynamics naturally captures the phenomenon of sparse coding and balanced inputs in a group of neurons. In contrast, in the traditional theory of a balanced network, the majority of neurons are balanced and thus fire actively. Therefore, in such a network, information can hardly be encoded by only a few of active neurons with the other neurons being quiescent. Note that, in order to achieve a balanced active core, our model assumes that the numbers of cortical excitatory and inhibitory inputs should be highly correlated, which has been supported by experimental observation (Liu, 2004).

### 3.4. Comparison With Previous Studies

Several studies have explored the dynamics of networks with heterogeneous connections. For example, in a recent study (Landau et al., 2016), there has been found a large fraction of neurons silent in the networks with sufficiently broad degree distributions. Moreover, it has been demonstrated that a heterogeneous network cannot reach a global balanced state in general when the cortical excitatory, cortical inhibitory and external in-degrees are uncorrelated, because some neurons either never fire or fire fairly regularly in the network (Landau et al., 2016). To achieve the global balanced state, the authors introduced adaptation, plasticity, or degree correlation into the network. In particular, by setting the number of cortical inhibitory input to be correlated with that of cortical excitatory input and with that of external excitatory input, the whole network will stay in the global balanced state (Landau et al., 2016), in which all neurons in the network receive balanced input and fire irregularly. Thus the balanced active group in such a case is the entire network. Different from their settings, here we set the correlation only between the numbers of cortical excitatory and inhibitory inputs, leaving the number of external input to be uncorrelated, and clearly show the mean firing rate of each neuron will exponentially decay with its in-degree, giving rise to the emergence of the active core in the network consisting of small-degree neurons. In addition, we further investigate the property of the active core, i.e., the subnetwork composed of the active neurons, and find that the balanced state does exist in the active core of the whole network. Our simulation shows that the balanced active core exists in a variety of networks with different degree distributions (scale-free, small-world, and broad Gaussian), while the cortical excitatory inputs are required to be correlated with cortical inhibitory inputs. Therefore, the existence of the active core seems not to be dependent on the degree distribution, but dependent on the correlation of cortical excitatory and inhibitory inputs. Moreover, by increasing the correlation between the number of external inputs and the number of cortical inputs to each neuron, the size of the active core will increase accordingly. Note that the increased correlation level has different effects on the active and quiescent groups. Intuitively, neurons in the active core are already balanced by its definition, thus the increase of correlation can only moderate the firing rate of these active neurons but have little effect on the size of the active core. However, for neurons in the quiescent group, such as neurons with degree *k*, the increase of correlation will drive these neurons toward the balanced state by satisfying the balanced condition, i.e., *kfν*_0_ + *kJ*_α*E*_*r*_*E*_ − *kJ*_α*I*_*r*_*I*_ ≈ 0. Therefore, as the correlation level increases, the size of the active core in the network will increase by recruiting more and more neurons that used to be in the quiescent group. Eventually, the size of the active core can be the same as the network size. We note that the balanced active core can also be found in networks with broad Gaussian distributions if the numbers of cortical excitatory input and cortical inhibitory input to each neuron are correlated, meanwhile they are uncorrelated with the number of the external input.

In another study (Argaman and Golomb, 2018), they have investigated a network of 150 inhibitory neurons in the barrel cortex with heterogeneous connections between each other, and neurons in this network receive heterogeneous excitatory inputs from thalamic neurons. The network does not have strong coupling and sparse connection. In addition, the number of inhibitory cortical input is set to be uncorrelated with the number of excitatory input. In this type of modestly-sized network, it has been found that the fraction of silent neurons is very small, and most of neurons seem to be in the balanced state. Here we have investigated strongly coupled but sparsely connected networks consisting of a large number of both excitatory and inhibitory neurons. With these different settings, we have shown that there is a large fraction of silent neurons in the network, and the balanced state only exists for a small fraction of neurons in the entire network, i.e., the active core.

Moreover, we have found that the degree distribution of the active core is close to the homogeneous connectivity structure. We note in passing that the emergence of the balanced active core does not naturally result from the high correlation between the numbers of the cortical excitatory inputs and the cortical inhibitory inputs. This can be illustrated by the following facts: first, large-degree neurons with such high correlation structure fail to reach the balanced state in general; second, small-degree neurons with such high correlation structure also fail to reach the balanced state if the network topology does not satisfy the third condition as discussed in section 4 (also shown in Figure 5). Another study has used a similar theoretical framework to investigate the effective gain in a heterogeneous network (Roxin, 2011). They also treated neurons with the same in-degree as one ensemble. However, they directly used the firing-rate-based neuron model, rather than deriving the FP equation as in our case. Moreover, the balanced property of the network has not been investigated in their work. In summary, the finding of the existence of the balanced active core embedded in a heterogeneous network distinguishes our work from several studies exploring the dynamics of networks with heterogeneous connections. Our work provides a potential dynamical scenario for the emergence of a balanced active core in a heterogeneous network in the brain.

## 4. Materials and Methods

### 4.1. Degree Distribution and Degree Correlation

In the study of networks, the degree of a node in a network is the number of connections it has to other nodes. For a directed network, nodes have two different degrees, the in-degree, which is the number of incoming edges to a node, and the out-degree, which is the number of outgoing edges from a node. In this work, we mainly focus on the in-degree distribution, and just use degree instead of in-degree in this work for ease of discussion. The degree distribution *P*(*k*) of a network is the probability of finding a *k*-node, where the *k*-node is a node of degree *k*. The degree distribution of a directed ER network follows the Poisson distribution, *P*(*k*) = λ^*k*^*e*^−λ^/*k*!, which can be approximated by a Gaussian distribution for large λ (λ ≫ 1), λ being the average degree of the network. The degree distribution of an SF network, by definition, follows a power-law distribution *P*(*k*) ∝ *k*^−γ^, γ being the decay exponent (Barabási et al., 1999).

Beyond the degree distribution, it is also important to characterize the degree-correlation between neighboring nodes for large networks of complex structures (Pastor-Satorras et al., 2001; Newman, 2003a). In general, a network may display degree-correlations if the wiring probability between the high- and low-degree nodes statistically significantly differs from the independent random wirings between nodes. In our work, the degree-correlation is quantified by the Pearson correlation coefficient between the in-degrees for pairs of nodes linked by a directed edge.

### 4.2. The Generation of Scale-Free Neuronal Networks

To generate an SF neuronal network, we first generate the in-degree (out-degree) of each neuron in the αth population based on the power-law distribution *P*_α, in_ (*P*_α, out_) for α = *E, I*. For ease of discussion, we set the first *N*_*E*_ nodes in the *N*-node network to be excitatory neurons, and those remaining are inhibitory. Then we generate *N* in/out-degree pairs (*k*_*i*_, *l*_*i*_) for each neuron, and calculate the sums $\sum_{i}k_{i}$ and $\sum_{i}l_{i}$. We use the method in Newman et al. (2001) to force the conservation of in-degree and out-degree, i.e., $\sum_{i}k_{i}=\sum_{j}l_{j}$. To be specific, when $\sum_{i}k_{i}\neq \sum_{j}l_{j}$, we randomly select a neuron *i* and regenerate a new pair of degree (*k*_*i*_, *l*_*i*_) from the corresponding degree distributions. We repeat the procedure until $\sum_{i}k_{i}=\sum_{j}l_{j}$. Then, we further define Γ_*i*_ as the ratio of the number of presynaptic excitatory neurons to that of presynaptic inhibitory neurons for the *i*th neuron, so that *k*_*i, E*_ = *k*_*i*_Γ_*i*_/(1 + Γ_*i*_) is the number of the excitatory incoming connections, and *k*_*i, I*_ = *k*_*i*_/(1 + Γ_*i*_) is the number of the inhibitory incoming connections for the *i*th neuron. Various levels of cross-correlations between the number of excitatory cortical inputs {*k*_*i, E*_} and the number of inhibitory cortical inputs {*k*_*i, I*_} can be obtained by choosing different values of {Γ_*i*_}. Finally, we make direct connections in the network according to {(*k*_*i, E*_, *k*_*i, I*_), *l*_*i*_} with the configuration model (Newman et al., 2001; Newman, 2003b). Note that the degrees of the connected nodes in such an SF network are uncorrelated (Aiello et al., 2000; Newman et al., 2001). To generate an SF network with degree correlation, we use a simple edge-node reshuffling strategy, which is a simplified version of the algorithm in Xulvi-Brunet and Sokolov (2005). In our simulations, unless otherwise specified, the decay exponent is chosen to be γ = 2.6 in our work, which is within the normal range of γ for real-world SF networks according to Barabási et al. (1999).

$C_{\alpha \beta }^{ij}$ is denoted as the element of the adjacency matrix with $C_{\alpha \beta }^{ij}=1$ if there is a directed edge from the *j*th neuron in the βth population to the *i*th neuron in the αth population, where α, β = *E, I*. If each neuron is connected, on average, to *K* presynaptic excitatory neurons and *K* presynaptic inhibitory neurons, because each neuron is connected to a large number of presynaptic neurons in the cortex (Braitenberg and Schüz, 1998), the value of *K* should be chosen sufficiently large to reflect this fact of connectivity. In addition, by electrophysiological recordings from cortical neurons, the probability of connection is shown to be often rather low, thus yielding a sparse network (Holmgren et al., 2003). Therefore, the value of *K* should be chosen to be much smaller than the size of the population. As the cells in the primary visual cortex of adult cats were found experimentally firing much more irregularly *in vivo* than the cells *in vitro* when the same stimulus was used (passing the same current through the electrode), fluctuations of the synaptic inputs are particularly important for irregular spiking (Holt et al., 1996). In light of this, we choose the scaling of the coupling strength to be of order $1/\sqrt{K}$, imparting fluctuations of order one to persist in the large-*K* limit in the total synaptic input to a neuron (van Vreeswijk and Sompolinsky, 1996; Vreeswijk and Sompolinsky, 1998; Vogels et al., 2005). We adopt this scaling for all the neuron models used in this work.

Next we explain how to find a power-law degree distribution with the decay exponent γ and the mean connectivity 2*K* for the generation of an SF network. Because the network size is always finite in numerical simulations, the degree of each neuron varies and has a lower bound denoted as *K*_0_ and an upper bound denoted as *K*_1_. Therefore, the power-law distribution takes the form as

$$\begin{array}{r} P\left(k\right)=Ck^{-\gamma }\;\text{for }k\in \left[K_{0},K_{1}\right], \end{array}$$

with a normalization constant *C*. By the definition of probability and its mean, we have

(7) $$\sum_{k=K_{0}}^{K_{1}}P(k)=1,\;\sum_{k=K_{0}}^{K_{1}}kP(k)=2K.$$

Intuitively, for fixed *K* and γ in an SF network, two parameters *K*_0_ and *K*_1_ cannot be simultaneously determined by Equation (7) since there are three unknowns, *C*, *K*_0_, and *K*_1_ and only two equations in Equation (7). This can be shown as follows.

1. For γ > 0 and γ ≠ 1, 2, Equation (7) can be approximately reformed as
  $$\begin{array}{l} \;1\approx \int_{K_{0}}^{K_{1}}P(k)dk=C\frac{K_{1}^{1-\gamma }-K_{0}^{1-\gamma }}{1-\gamma },\\ 2K\approx \int_{K_{0}}^{K_{1}}kP(k)dk=C\frac{K_{1}^{2-\gamma }-K_{0}^{2-\gamma }}{2-\gamma }. \end{array}$$
  Subsequently, we can obtain the following relationship
  (8) $$\begin{array}{r} 2K\approx \frac{1-\gamma }{2-\gamma }\cdot \frac{{\left(K_{1}/K_{0}\right)}^{2-\gamma }-1}{{\left(K_{1}/K_{0}\right)}^{1-\gamma }-1}K_{0}. \end{array}$$
2. For γ = 1, Equation (7) can be calculated that
  $$\begin{array}{l} \;1\approx \int_{K_{0}}^{K_{1}}P(k)dk=C\ln(K_{1}/K_{0}),\\ 2K\approx \int_{K_{0}}^{K_{1}}kP(k)dk=C(K_{1}-K_{0}). \end{array}$$
  Then, we can obtain
  (9) $$2K\approx \frac{K_{1}/K_{0}-1}{\ln(K_{1}/K_{0})}K_{0}.$$
3. For γ = 2, it can be calculated that
  $$\begin{array}{l} 1\approx \int_{K_{0}}^{K_{1}}P(k)dk=C\left(\frac{1}{K_{0}}-\frac{1}{K_{1}}\right),\\ 2K\approx \int_{K_{0}}^{K_{1}}kP(k)dk=\ln(K_{1}/K_{0}). \end{array}$$
  Similarly, we have
  (10) $$\begin{array}{r} 2K\approx \frac{\ln\left(K_{1}/K_{0}\right)}{1-K_{0}/K_{1}}K_{0}. \end{array}$$

Then, given the value of *K* and γ, we can choose proper *K*_0_ and *K*_1_ following one of Equations (8)–(10) to ensure that the balanced condition (3) holds. Since the starting point of the power-law distribution of the degree normalized by the network size from an experimental observation is about 0.95% (Bonifazi et al., 2009), we choose $K_{0}\approx 380=0.95\%\times \left(4\times 10^{4}\right)$ accordingly in many of our simulations, where 4 × 10^4^ is the network size. The value of *K*_0_ is set to be different from 380 only when we investigate the effect of the network size in Figure 4B. Note that *K*_1_ cannot be larger than the network size.

### 4.3. The Current-Based I&F Model With Delta-Pulse Coupling

In our work, the sub-threshold membrane potential of an I&F neuron in a population obeys the following dynamics (Dayan et al., 2001; Newhall et al., 2010; Zhou et al., 2010)

(11) $$\begin{array}{r} \frac{dv_{\alpha }^{i}}{dt}=-g_{L}\left(v_{\alpha }^{i}-\epsilon_{R}\right)+I_{\alpha }^{i}\left(t\right), \end{array}$$

where $v_{\alpha }^{i}$ is the membrane potential of the *i*th neuron in the αth population (α = *E, I*), *g*_*L*_ is the leakage conductance, ϵ_*R*_ is the resting voltage, and $I_{\alpha }^{i}\left(t\right)$ is the driving current. The voltage $v_{\alpha }^{i}$ evolves according to Equation (11) while it remains below the firing threshold ϵ_*T*_. When $v_{\alpha }^{i}$ reaches ϵ_*T*_, the *i*th neuron is said to fire a spike, and $v_{\alpha }^{i}$ is set to the value of the reset voltage ϵ_*R*_. Upon resetting, $v_{\alpha }^{i}$ is governed by Equation (11) again. At the same time, appropriate currents induced by the spike are injected into all other postsynaptic neurons. We use physiological values for the parameters *g*_*L*_ = 50 s^−1^, ϵ_*R*_ = −70 mV and ϵ_*T*_ = −55 mV. Upon non-dimensionalization, we have normalized ϵ_*T*_ = 1.0 and ϵ_*R*_ = 0.0.

The instantaneous current $I_{\alpha }^{i}\left(t\right)$ injected into the *i*th neuron of the αth population has the following form $I_{\alpha }^{i}\left(t\right)=I_{\alpha E}^{i}\left(t\right)+I_{\alpha I}^{i}\left(t\right)$, where $I_{\alpha I}^{i}(t)=-J_{\alpha I}\sum_{j=1}^{N_{I}}C_{\alpha I}^{ij}\sum_{s}\delta (t-\tau_{js}^{I})$ is the inhibitory input, whereas $I_{\alpha E}^{i}(t)=f_{\alpha }\sum_{s}\delta (t-\varsigma_{is}^{\alpha })+J_{\alpha E}\sum_{j=1}^{N_{E}}C_{\alpha E}^{ij}\sum_{s}\delta (t-\tau_{js}^{E})$ is the excitatory input — δ(·) is the Dirac delta function, *J*_αβ_ is the coupling strength from the βth population to the αth population (α, β = *E, I*), and *f*_α_ is the strength of the external Poisson input to the αth population. The first term in $I_{\alpha E}^{i}\left(t\right)$ corresponds to the current from the external input. The external input of the *i*th neuron in the αth population is modeled by a Poisson process $\left\{\varsigma_{is}^{\alpha }\right\}$ with rate ν_α_. At the time, $t=\varsigma_{is}^{\alpha }$, of the *s*th input spike to the *i*th neuron in the αth population, the neuron's voltage jumps by the amount of *f*_α_. The second term in $I_{\alpha E}^{i}\left(t\right)$ and the term in $I_{\alpha I}^{i}\left(t\right)$ correspond to the currents induced by the coupled neurons in the excitatory and inhibitory populations in the network, in which $\left\{\tau_{js}^{E}\right\}$ is the spike train from the *j*th neuron in the excitatory population, $\left\{\tau_{js}^{I}\right\}$ is the spike train from the *j*th neuron in the inhibitory population, and *s* denotes the *s*th spike in the train.

In the simulation, the values of parameters in the model are set as follows: $J_{EE}=J_{IE}=1.0/\sqrt{K}$, $J_{II}=1.8/\sqrt{K},J_{EI}=2.0/\sqrt{K}$, $f_{E}=f_{I}=1.0/\sqrt{K}$, and ν_*E*_ = ν_0_*K*, ν_*I*_ = 0.8ν_0_*K*. We vary the value of ν_0_ to control the rate of the external input. To perform the numerical simulation of this I&F model, we use an event-driven scheme (Brette et al., 2007), with which the numerical results of dynamics can be obtained up to the machine accuracy.

### 4.4. Fokker-Planck Equation for a Single Neuron

Under a Poisson external input, the spiking events of a neuron in the network, in general, are not Poissonian, i.e., $\left\{\tau_{js}^{E}\right\}$ and $\left\{\tau_{js}^{I}\right\}$ in the current $I_{\alpha }^{i}\left(t\right)$ are not a Poisson process for a fixed neuron *j*. However, the input to the *i*th neuron is a spike train summed over output spike trains from many other neurons in the network. If the firing event of each neuron is statistically independent of one another, then the spike train obtained by summing over a large number of output spike trains of neurons asymptotically tends to a Poisson process (Cinlar, 1972). In a balanced network, the firing event of each neuron is extremely weakly correlated with, and nearly independent of, other neurons (Vreeswijk and Sompolinsky, 1998). Therefore, for each neuron, the summed incoming spikes from its presynaptic neurons can be approximated by a Poisson train. Under the Poisson approximation, we can obtain the Fokker-Planck (FP) equation corresponding to Equation (11) for each neuron in the population (Cai et al., 2006). For the *i*th neuron in the αth population, we have

(12) $$\frac{\partial }{\partial t}\rho_{\alpha }^{i}=\frac{\partial }{\partial v}\left[(g_{L}v-\mu_{\alpha }^{i})\rho_{\alpha }^{i}\right]+\frac{{\sigma_{\alpha }^{i}}^{2}}{2}\frac{\partial^{2}}{\partial v^{2}}\rho_{\alpha }^{i},$$

where $\rho_{\alpha }^{i}\left(v,t\right)$ is the probability density at time *t* of finding the membrane potential at *v* of the *i*th neuron in the αth population. Here $\mu_{\alpha }^{i}$ is the mean total input,

(13) $$\begin{array}{r} \mu_{\alpha }^{i}=f_{\alpha }\nu_{\alpha }+J_{\alpha E}\nu_{\alpha E}^{i}-J_{\alpha I}\nu_{\alpha I}^{i}, \end{array}$$

and ${\left(\sigma_{\alpha }^{i}\right)}^{2}$ is the strength of fluctuations of the total input,

(14) $$\begin{array}{r} {\left(\sigma_{\alpha }^{i}\right)}^{2}=f_{\alpha }^{2}\nu_{\alpha }+J_{\alpha E}^{2}\nu_{\alpha E}^{i}+J_{\alpha I}^{2}\nu_{\alpha I}^{i}. \end{array}$$

Note that $\nu_{\alpha E}^{i}$ and $\nu_{\alpha I}^{i}$ are the rates of the summed respective excitatory and inhibitory inputs from other neurons in the network, *f*_α_ and ν_α_ are the strength and rate of the external Poisson input to the αth population, respectively.

Equation (12) can be cast into the conservation form $\frac{\partial }{\partial t}\rho_{\alpha }^{i}\left(v,t\right)+\frac{\partial }{\partial v}S_{\alpha }^{i}\left(v,t\right)=0$, with $\;S_{\alpha }^{i}(v,t)=-\frac{{\left(\sigma_{\alpha }^{i}\right)}^{2}}{2}\frac{\partial }{\partial v}\rho_{\alpha }^{i}-g_{L}\left(v-\frac{\mu_{\alpha }^{i}}{g_{L}}\right)\rho_{\alpha }^{i}$ being the probability density flux through *v* at time *t*. For Equation (12), we need to specify boundary conditions at *v* = −∞, the reset potential ϵ_*R*_ , and the threshold ϵ_*T*_. The probability flux through ϵ_*T*_ gives the instantaneous firing rate at *t*, $m_{\alpha }^{i}\left(t\right)=S_{\alpha }^{i}\left(\epsilon_{T},t\right)$. For the I&F neuron, its membrane potential cannot exceed the threshold, therefore, $\rho_{\alpha }^{i}\left(v,t\right)=0$ for *v* ≥ ϵ_*T*_. At the reset potential *v* = ϵ_*R*_ , there is a probability flux coming from the neuron that just crosses the threshold: what goes out at time *t* at the threshold must come back at time *t* at the reset potential, thus $S_{\alpha }^{i}\left(\epsilon_{R}^{+},t\right)-S_{\alpha }^{i}\left(\epsilon_{R}^{-},t\right)=m_{\alpha }^{i}\left(t\right)$. The natural boundary condition at *v* = −∞ is $\rho_{\alpha }^{i}$ tends sufficiently rapidly toward zero to be integrable, $\underset{v\to -\infty }{\lim}\rho_{\alpha }^{i}\left(v,t\right)=0$ and $\underset{v\to -\infty }{\lim}v\rho_{\alpha }^{i}\left(v,t\right)=0$. By definition, $\rho_{\alpha }^{i}\left(v,t\right)$ satisfies the normalization condition $\int_{-\infty }^{V_{T}}\rho_{\alpha }^{i}(v,t)dv=1$.

The stationary solution of Equation (12) can be obtained as Brunel (2000)

(15) $$\begin{array}{l} \rho_{\alpha ,k}^{i}=\frac{2m_{\alpha }^{i}}{{\left(\sigma_{\alpha }^{i}\right)}^{2}}\;\exp\;\left(-\frac{g_{L}}{{\left(\sigma_{\alpha }^{i}\right)}^{2}}{\left(v-\mu_{\alpha }^{i}\right)}^{2}\right)\\ \;\int_{v}^{\epsilon_{T}}\Theta (u-\epsilon_{R})\exp\left(\frac{g_{L}}{{\left(\sigma_{\alpha }^{i}\right)}^{2}}{\left(u-\mu_{\alpha }^{i}\right)}^{2}\right)du. \end{array}$$

Furthermore, by using the normalization condition, the firing rate $m_{\alpha }^{i}$ can be obtained as

(16) $$m_{\alpha }^{i}=g_{L}{\left\{\sqrt{\pi }\int_{\frac{\epsilon_{R}-\mu_{\alpha }^{i}/g_{L}}{\sigma_{\alpha }^{i}/\sqrt{g_{L}}}}^{\frac{\epsilon_{T}-\mu_{\alpha }^{i}/g_{L}}{\sigma_{\alpha }^{i}/\sqrt{g_{L}}}}\exp(x^{2})\left[1+\text{erf}(x)\right]dx\;\right\}}^{-1},$$

where erf(*x*) is the error function.

### 4.5. Fokker-Planck Equation for a Homogeneous Network

For the balanced state in homogeneous neuronal networks, one can reach a probabilistic characterization of the network beyond the dynamics of a single neuron. Because each neuron in the balanced state of a homogeneous network can be regarded as nearly statistically identical in a particular population, the input spike train of each neuron, which is summed from all presynaptic neurons, is Poisson with rate *K*_α_*m*_α_(*t*), by noting that each neuron has *K*_*E*_ presynaptic excitatory neurons and *K*_*I*_ presynaptic inhibitory neurons on average. Here *m*_α_(*t*) is the population-averaged firing rate for a neuron in the αth population, α = *E, I*. Then, one can obtain

(17) $$\begin{array}{r} \frac{\partial }{\partial t}\rho_{\alpha }\left(v,t\right)+\frac{\partial }{\partial v}S_{\alpha }\left(v,t\right)=0, \end{array}$$

where ρ_α_(*v, t*) is the probability of finding a neuron in the αth population whose membrane potential is *v* at time *t* (Brunel, 2000), and the probability density flux $S_{\alpha }\left(v,t\right)=-\frac{{\sigma_{\alpha }}^{2}}{2}\frac{\partial }{\partial v}\rho_{\alpha }$ $-g_{L}\left(v-\frac{\mu_{\alpha }}{g_{L}}\right)\rho_{\alpha }$, where the input is characterized by μ_α_ = *f*_α_ν_α_+*J*_α*E*_*K*_*E*_*m*_*E*_−*J*_α*I*_*K*_*I*_*m*_*I*_ and ${\sigma_{\alpha }}^{2}=f_{\alpha }^{2}\nu_{\alpha }+J_{\alpha E}^{2}K_{E}m_{E}+J_{\alpha I}^{2}K_{I}m_{I}$. By the same argument for Equation (12), the boundary conditions for Equation (17) can be similarly obtained.

Similar to the single-neuron case, here the mean firing rate over the neuronal population can be obtained in a self-consistent way as

(18) $$m_{\alpha }=g_{L}{\left\{\sqrt{\pi }\int_{\frac{\epsilon_{R}-\mu_{\alpha }/g_{L}}{\sigma_{\alpha }/\sqrt{g_{L}}}}^{\frac{\epsilon_{T}-\mu_{\alpha }/g_{L}}{\sigma_{\alpha }/\sqrt{g_{L}}}}\exp(x^{2})\left[1+\text{erf}(x)\right]dx\right\}}^{-1}.$$

### 4.6. Fokker-Planck Equation for a Scale-Free Network

One can further derive the FP equations for an SF network with the following structural property — for each neuron in the network, the ratio of the number of its presynaptic excitatory neurons to the number of its presynaptic inhibitory neurons is almost a constant across the population. By denoting this constant ratio as Γ (Γ = 1 for the SF networks in our simulations), and treating all the neurons with the same number of presynaptic neurons as one ensemble, we can derive the FP equation for the *k*th ensemble (the neuron ensemble with *k* presynaptic neurons) in the αth population,

$$\frac{\partial }{\partial t}\rho_{\alpha }^{k}=\frac{\partial }{\partial v}\left[(g_{L}v-\mu_{\alpha }^{k})\rho_{\alpha }^{k}\right]+\frac{{\left(\sigma_{\alpha }^{k}\right)}^{2}}{2}\frac{\partial^{2}}{\partial v^{2}}\rho_{\alpha }^{k},$$

where $\mu_{\alpha }^{k}$ is the average total input

(19) $$\begin{array}{r} \mu_{\alpha }^{k}=f_{\alpha }\nu_{\alpha }+kJ_{\alpha E}\frac{\Gamma }{1+\Gamma }r_{E}^{k}-kJ_{\alpha I}\frac{1}{1+\Gamma }r_{I}^{k}, \end{array}$$

and ${\left(\sigma_{\alpha }^{k}\right)}^{2}$ describes the strength of fluctuations of the total input

(20) $$\begin{array}{r} {\left(\sigma_{\alpha }^{k}\right)}^{2}={f_{\alpha }}^{2}\nu_{\alpha }+k{J_{\alpha E}}^{2}\frac{\Gamma }{1+\Gamma }r_{E}^{k}+k{J_{\alpha I}}^{2}\frac{1}{1+\Gamma }r_{I}^{k}. \end{array}$$

Here $r_{E}^{k}=\int_{K_{0}}^{K_{1}}T(n|k)m_{E}^{n}dn$ and $r_{I}^{k}=\int_{K_{0}}^{K_{1}}T(n|k)m_{I}^{n}dn$ are the presynaptic excitatory and inhibitory neurons' mean firing rates for the *k*th ensemble respectively, *T*(*n*|*k*) is the conditional probability of finding a directed connection that originates from an *n*-node (a neuron with *n* presynaptic neurons) given that it ends at a *k*-node (a neuron with *k* presynaptic neurons). And *K*_0_ (*K*_1_) denotes the smallest (largest) degree. In the stationary state, one can obtain the firing rate of neurons in the *k*-ensemble as

(21) $$m_{\alpha }^{k}=g_{L}{\left\{\sqrt{\pi }\int_{\frac{\epsilon_{R}-\mu_{\alpha }^{k}/g_{L}}{\sigma_{\alpha }^{k}/\sqrt{g_{L}}}}^{\frac{\epsilon_{T}-\mu_{\alpha }^{k}/g_{L}}{\sigma_{\alpha }^{k}/\sqrt{g_{L}}}}\exp(x^{2})\left[1+\text{erf}(x)\right]dx\right\}}^{-1}.$$

When there is no degree correlation between each node, the conditional probability can be calculated as *T*(*n*|*k*) = *nP*(*n*)/(2*K*) independent of *k*, where *P*(*n*) is the power-law degree distribution. Then according to Equations (19–20), $\mu_{\alpha }^{k}$ decreases linearly with the degree *k* because the network is inhibition dominant, while ${\left(\sigma_{\alpha }^{k}\right)}^{2}$ increases linearly with the degree *k*. Moreover, for neurons with a large number of presynaptic connections, i.e., large *k*, one can find that $\mu_{\alpha }^{k}\sim -k$ and $\sigma_{\alpha }^{k}\sim \sqrt{k}$. Therefore, $\epsilon_{R}-\mu_{\alpha }^{k}/g_{L}\gg \sigma_{\alpha }^{k}/\sqrt{g_{L}}$, and $\epsilon_{T}-\mu_{\alpha }^{k}/g_{L}\gg \sigma_{\alpha }^{k}/\sqrt{g_{L}}$. The mean firing rate can thus be further approximated as

(22) $$m_{\alpha }^{k}\approx \frac{g_{L}(\epsilon_{T}-\mu_{\alpha }^{k}/g_{L})}{\sqrt{\pi }(\epsilon_{T}-\epsilon_{R})}\exp\;\left\{-\;{\left(\frac{\epsilon_{T}-\mu_{\alpha }^{k}/g_{L}}{\sigma_{\alpha }^{k}/\sqrt{g_{L}}}\right)}^{2}\right\}.$$

Because $\mu_{\alpha }^{k}\sim -k$ and $\sigma_{\alpha }^{k}\sim \sqrt{k}$, according to Equation (22), the firing rate of a neuron $m_{\alpha }^{k}$ in the *k*th ensemble decays exponentially with *k*. Consequently, neurons with a sufficiently large degree will possess a very low firing rate which can barely be detected in numerical results with a finite simulation time, thus they will be classified to the quiescent group.

We comment that, for a homogeneous network with a broad degree distribution, the group of quiescent neurons also exists. As the width of the degree distribution becomes broader, the number of the quiescent neurons becomes larger. This phenomenon can also be explained from the result of the corresponding FP analysis in Roxin et al. (2011).

## Author Contributions

QG, SL, WD, DZ, and DC conceived and designed the research, performed experiments and analyzed data, and wrote the paper.

### Conflict of Interest Statement

The authors declare that the research was conducted in the absence of any commercial or financial relationships that could be construed as a potential conflict of interest.
