# Supplementary material for: Balanced Active Core in Heterogeneous Neuronal Networks
Source: Front Comput Neurosci. 2019 Jan 29;12:109. doi: 10.3389/fncom.2018.00109 (PMC6360995; doi:10.3389/fncom.2018.00109)
Supplement: Supplementary file 1 [file Presentation_1.pdf]

Supplementary Material: Balanced active core in heterogeneous neuronal networks

1 SUPPLEMENTARY FIGURES

S1 Fig

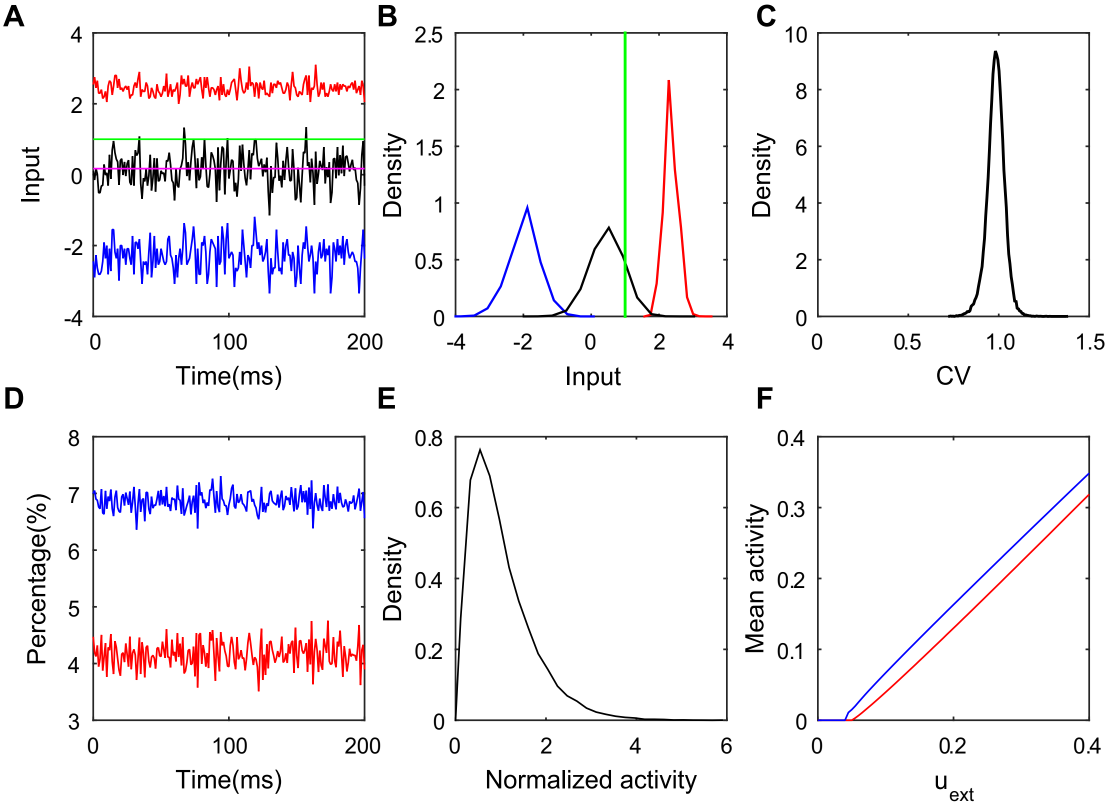

Figure S1. Properties of a balanced network of binary neurons with homogeneous topology. (A): The balanced excitatory and inhibitory inputs into a sample neuron (transient dynamics have been removed). The magnitudes of excitatory (red) and inhibitory (blue) inputs are greater than the firing threshold (green), whereas the total input (black) crosses the threshold stochastically with its mean (magenta, the value is 0.17) remaining below the threshold; **(B)**: The probability density functions of the excitatory (red), inhibitory (blue) and total (black) inputs for the sample neuron in panel **(A)**. The green line stands for the threshold; **(C)**: The distribution of the CV value for the ISIs of each neuron. The distribution is far from zero, indicating high firing irregularity of all neurons; **(D)**: The population-averaged excitatory (red) and inhibitory (blue) activities. The population-averaged activity is the percentage of active neurons in the population at any given time (transient dynamics have been removed); **(E)**: The distribution of the mean firing activity of each neuron; **(F)**: The mean activity as a linear function of the external input parameter u_{ext} for the excitatory population (red solid line) and the inhibitory population (blue solid line). Here, $N_E = N_I = 2 \times 10^4$ and $K = 400$. In panels **(A)-(E)**, $u_{\text{ext}} = 0.1$.

The properties of homogeneous balanced network The properties here summarized from the binary neuronal network Vreeswijk and Sompolinsky (1998). In the binary model, the activity of the ith neuron in the αth population ($\alpha = E, I$) is described by the binary variable $\sigma_\alpha^i(t + \Delta t) = \Theta(u_\alpha^i(t))$, where $\Theta(x)$ is the Heaviside function, and $u_\alpha^i(t)$ equals the total synaptic input projecting into the ith neuron in the αth population above the threshold θ_α at time t,

$$u_\alpha^i(t) = I_{\alpha E}^i(t) + I_{\alpha I}^i(t) - \theta_\alpha, \tag{S1}$$

where $I_{\alpha E}^i(t) = J_{\alpha E} \sum_{j=1}^{N_E} C_{\alpha E}^{ij} \sigma_E^j(t) + u_\alpha^0$ and $I_{\alpha I}^i(t) = -J_{\alpha I} \sum_{j=1}^{N_I} C_{\alpha I}^{ij} \sigma_I^j(t)$, u_α^0 is the constant external input to the αth population, and $J_{\alpha\beta}$ describes the coupling strength from the βth population to the αth population ($\alpha, \beta = E, I$), which is scaled as $1/\sqrt{K}$ as described above.

In our simulation, the values of the parameters chosen for the binary model are as follows : $J_{EE} = J_{IE} = 1.0/\sqrt{K}$, $J_{II} = 1.8/\sqrt{K}$, $J_{EI} = 2.0/\sqrt{K}$, $\theta_E = 1.0$, $\theta_I = 0.7$, $u_E^0 = u_{\text{ext}}\sqrt{K}$, $u_I^0 = 0.8u_{\text{ext}}\sqrt{K}$, where u_{ext} controls the magnitude of the external input.

For the balanced state in the ER network, one can obtain the mean population rate as Vreeswijk and Sompolinsky (1998)

$$m_E = \frac{1}{K} \frac{J_{II} u_E^0 - J_{EI} u_I^0}{J_{EI} J_{IE} - J_{II} J_{EE}}, \quad m_I = \frac{1}{K} \frac{J_{IE} u_E^0 - J_{EE} u_I^0}{J_{EI} J_{IE} - J_{II} J_{EE}}. \tag{S2}$$

As mentioned above, both u_E^0 and u_I^0 are proportional to u_{ext}. Thus, Eq. (S2) exhibits a linear relation between the mean activity rate m_k and the external-input magnitude u_{ext}. Next, we enumerate all five hallmarks of a balanced network as following:

1. **Balanced net input:** As illustrated in figure. S1A in the Supplemental Data, the magnitude of either the excitatory or the inhibitory input to a neuron is much higher than the neuronal firing threshold. However, because of the dynamically induced cancellation between the excitation and inhibition, the total input has its mean staying below the threshold all the time while its fluctuations stochastically driving the membrane potential across the threshold. Figure. S1B in the Supplemental Data illustrates the same phenomena from the viewpoint of distribution. The mean excitatory and inhibitory inputs of each neuron are large, while the mean total input of each neuron remains below the threshold;
2. **Irregular activity:** To quantify the irregular firing of a neuron in the balanced state, we use the coefficient of variation (CV), the ratio of standard deviation to mean, of the distribution of inter-spike-interval (ISI) for each neuron. Clearly, if CV $= 0$, the neuron fires regularly. As captured in figure. S1C in the Supplemental Data, the distribution of the CV value is significantly far from zero for the balanced state;
3. **Stationary population-averaged activity:** We next examine the population-averaged activity, $m_k(t) = \sum_{i=1}^{N_k} \sigma_k^i(t)/N_k$ for $k = E, I$, which is the percentage of active neurons in the population at any given time. For a balanced state, as shown in figure. S1D in the Supplemental Data, the population-averaged activity almost stays constant over time as a consequence of the entire system reaching a stationary state;

4. **Heterogeneity of firing rate:** As shown in figure. S1E in the Supplemental Data, the activity of neurons is rather heterogeneous, which is characterized by a broad and rather skewed distribution of single-neuron firing rate in the network;
5. **Linear response:** As exemplified in figure. S1F in the Supplemental Data, a balanced state possesses the linear response of the mean activity of both the excitatory and inhibitory populations to the external input despite the nonlinear governing dynamics of each individual neuron. As noted above, in the large-K limit, this defining property of linearity can be captured by Eq. (S2) under the mean-field approximation.

S2 Fig

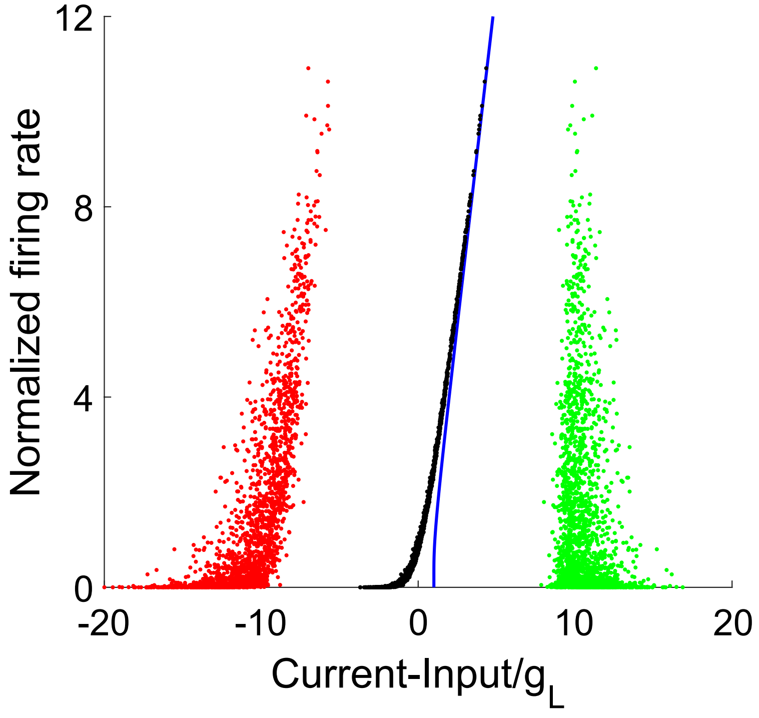

Figure S2. Firing rate of each firing neuron as a function of its inputs. The firing rates of individual neurons vs. the time averaged inhibitory inputs (red dots), excitatory inputs (green dots), net inputs (black dots). Each dot in each color corresponds one firing neuron. The blue curve is calculated when an I&F neuron receives a constant current with various amplitudes. Note that we only plot neurons that fire in simulations and some of the neurons here with small mean input current have small but non-zero firing rates.

Firing rate of each firing neuron as a function of its inputs. The time-averaged inhibitory and excitatory inputs vary from neuron to neuron, with their population average much larger than the threshold. We can find that the firing rate of each firing neuron is linearly correlated with the net input.

S3 Fig

Fluctuations of net input current as a function of network size. The population-averaged standard deviation (Std) of the net input current is normalized by the leak conductance g_L. The values nearly stay at order one and do not decay with the network size N.

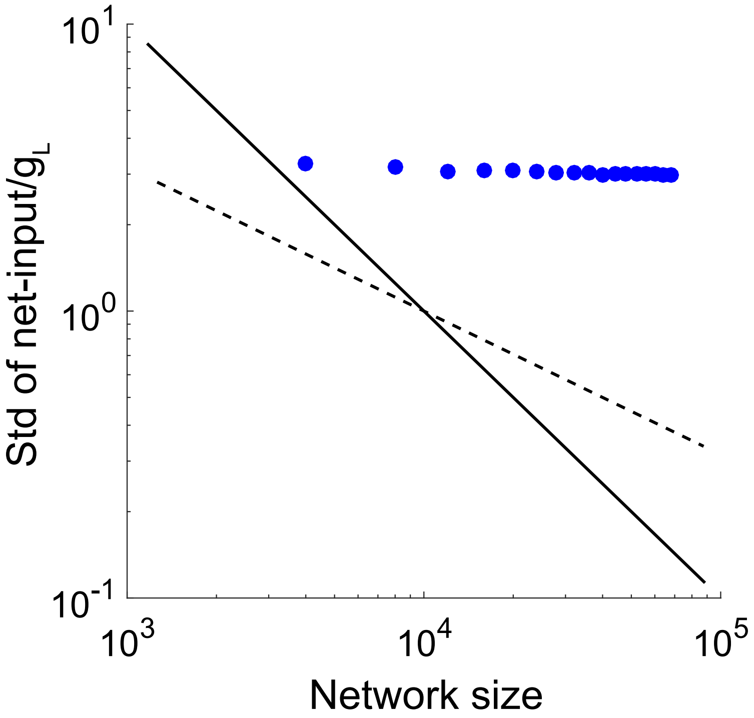

Figure S3. Fluctuations of net input current as a function of network size. The blue dots are the population-averaged standard deviation (Std) of the net input current (normalized by g_L) obtained from simulations. The black solid line indicates the decay rate of N^{-1}. The black dashed line indicates the decay rate of $N^{-0.5}$. Here, the average connectivity is $K = 0.05 * N/2$.

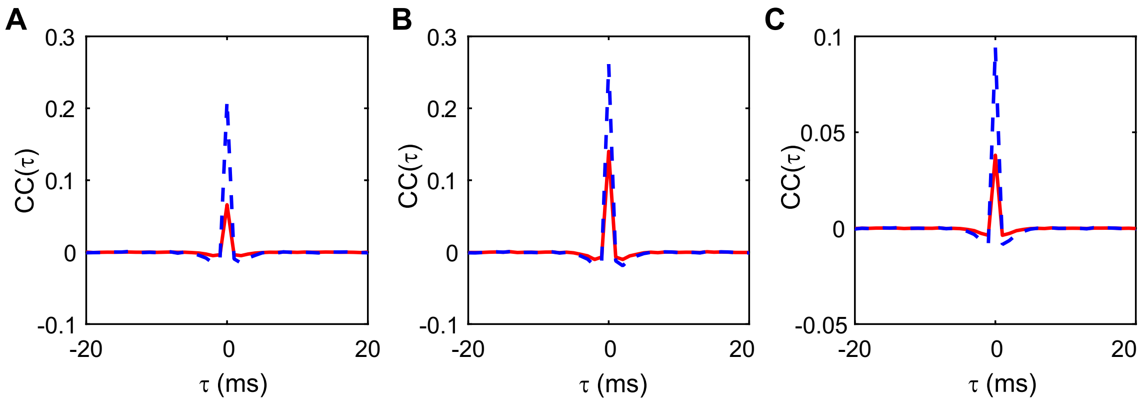

Figure S4. Cross-correlations (CC) between inputs. **(A)**: The average cross-correlation between the excitatory and inhibitory synaptic inputs for neurons in the active group (red solid line) and neurons in the quiescent group (blue dashed line). **(B)**-**(C)**: The average cross-correlation between two inputs of identical type across all pairs of neurons in the active group (red) and the quiescent group (blue). **(B)** is for excitatory inputs and **(C)** is for inhibitory inputs.

S4 Fig

Cross-correlations (CC) between synaptic inputs. Here we introduce how the average cross-correlation is calculated.

As discussed in Sec. *Materials and Methods*, the synaptic inputs to the ith neuron in the αth population is $I^i_\alpha(t) = I^i_{\alpha I}(t) + I^i_{\alpha E}(t)$, where $I^i_{\alpha I}(t) = -J_{\alpha I}\sum_{j=1}^{N_I} C^{ij}_{\alpha I}\sum_s \delta(t-\tau^I_{js})$ is the inhibitory input, and $I^i_{\alpha E}(t) = f_\alpha \sum_s \delta(t-\varsigma^\alpha_{is}) + J_{\alpha E}\sum_{j=1}^{N_E} C^{ij}_{\alpha E}\sum_s \delta(t-\tau^E_{js})$ is the excitatory input. Then the CC between the excitatory and inhibitory synaptic inputs of the ith neuron in the αth population can be calculated as

$$CC^i_\alpha(\tau) = \frac{\langle (I^i_{\alpha E}(t) - \langle I^i_{\alpha E}(t)\rangle)(I^i_{\alpha I}(t+\tau) - \langle I^i_{\alpha I}(t)\rangle)\rangle}{\sqrt{\langle (I^i_{\alpha E}(t) - \langle I^i_{\alpha E}(t)\rangle)^2\rangle\langle (I^i_{\alpha I}(t) - \langle I^i_{\alpha I}(t)\rangle)^2\rangle}}, \tag{S3}$$

and The CC between the excitatory (inhibitory) synaptic inputs of the ith neuron in the αth population and the jth neuron in the βth population can be calculated as

$$\begin{aligned} CC^{ij}_{\alpha\beta,E}(\tau) &= \frac{\langle (I^i_{\alpha E}(t) - \langle I^i_{\alpha E}(t)\rangle)(I^j_{\beta E}(t+\tau) - \langle I^j_{\beta E}(t)\rangle)\rangle}{\sqrt{\langle (I^i_{\alpha E}(t) - \langle I^i_{\alpha E}(t)\rangle)^2\rangle\langle (I^j_{\beta E}(t) - \langle I^j_{\beta E}(t)\rangle)^2\rangle}}, \\ CC^{ij}_{\alpha\beta,I}(\tau) &= \frac{\langle (I^i_{\alpha I}(t) - \langle I^i_{\alpha I}(t)\rangle)(I^j_{\beta I}(t+\tau) - \langle I^j_{\beta I}(t)\rangle)\rangle}{\sqrt{\langle (I^i_{\alpha I}(t) - \langle I^i_{\alpha I}(t)\rangle)^2\rangle\langle (I^j_{\beta I}(t) - \langle I^j_{\beta I}(t)\rangle)^2\rangle}}, \end{aligned} \tag{S4}$$

where the bracket $\langle\cdot\rangle$ indicates a time average. The average CCs are can then be obtained by averaging CC for each pair of inputs over the active and quiescent groups respectively. In our simulations, CCs are calculated from the average of $n = 300$ randomly chosen neurons in each subgroup.

S5 Fig

The influence of the E-I input strength ratio ϕ and the decay exponent of degree distribution γ on the properties of the balanced core. From the results, The value of γ and ϕ have a strong influence on the size of the active core. The degree distribution of the core can be well captured by Eq. (3) for various γ. We notice that the size of the active core is approximately equal to the probability of finding an active neuron among all presynaptic neurons for each neuron p as observed in our simulations. Therefore, figure. S5A in the Supplemental Data also illustrates the dependence of the probability p on ϕ.

S6 Fig

Properties of a balanced SF neuronal network with degree-correlation. The degree correlation coefficient of SF network is $\rho = 0.03$. Our results in figure. S6 in the Supplemental Data show that there exists a balanced state in the correlated SF neuronal network.

S7 Fig

The active core in SF neuronal networks with different degree correlations. The measured distribution of the active core in the SF neuronal network with different degree correlations, ranging from $\rho = -0.3$ to $\rho = +0.31$, is similar to that of an ER network. Note that the active core is the subnetwork consisting of all the active neurons and the connectivity structure of these active neurons.

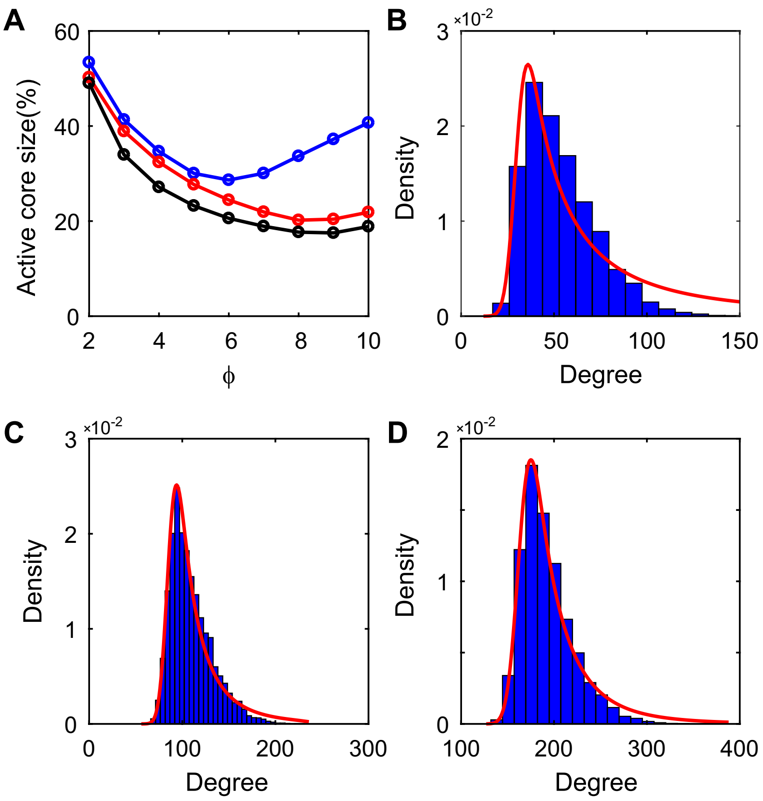

Figure S5. The influence of the E-I input strength ratio ϕ and the decay exponent of degree distribution γ on the properties of the balanced core. (A) The size of the active core (or approximately the probability p of finding an active neuron among all presynaptic neurons for each neuron) as a function of ϕ. Color codes various γ values: $\gamma = 3.0$ (blue), $\gamma = 2.0$ (red), and $\gamma = 1.0$ (black). **(B)-(D)** The degree distribution of the active core in different SF networks with different decay exponents γ for fixed $\phi = 3.0$. **(B)** $\gamma = 1.0$; **(C)** $\gamma = 2.0$; **(D)** $\gamma = 3.0$. Numerical results (blue bars) can be well fitted by our prediction (Eq. (3), red line).

S8 Fig

Binary model with SF connectivity. We can find the balanced state in the SF network containing simple binary neurons.

S9 Fig

Smooth-current-based I&F neuronal network with SF connectivity. In this model we use $\alpha(t) = \Big(\exp\big(-\frac{t}{\tau_r^k}\big) - \exp\big(-\frac{t}{\tau_d^k}\big)\Big)/(\tau_r^k - \tau_d^k)$ (for $k = E, I$) as the smooth current input instead of delta-pulse current input and the sub-threshold membrane potential of a neuron still obeys Eq. (8). Here, $\tau_r^E = \tau_r^I = 1\,\mathrm{ms}$, $\tau_d^E = 5\,\mathrm{ms}$, $\tau_d^I = 10\,\mathrm{ms}$.

S10 Fig

The degree distribution of the active core for the SF network consisting of binary neurons and smooth-current-based I&F neurons. The measured distribution of the active core from the SF network consisting either of binary neurons or of smooth-current-based I&F neurons is similar to that of an ER network.

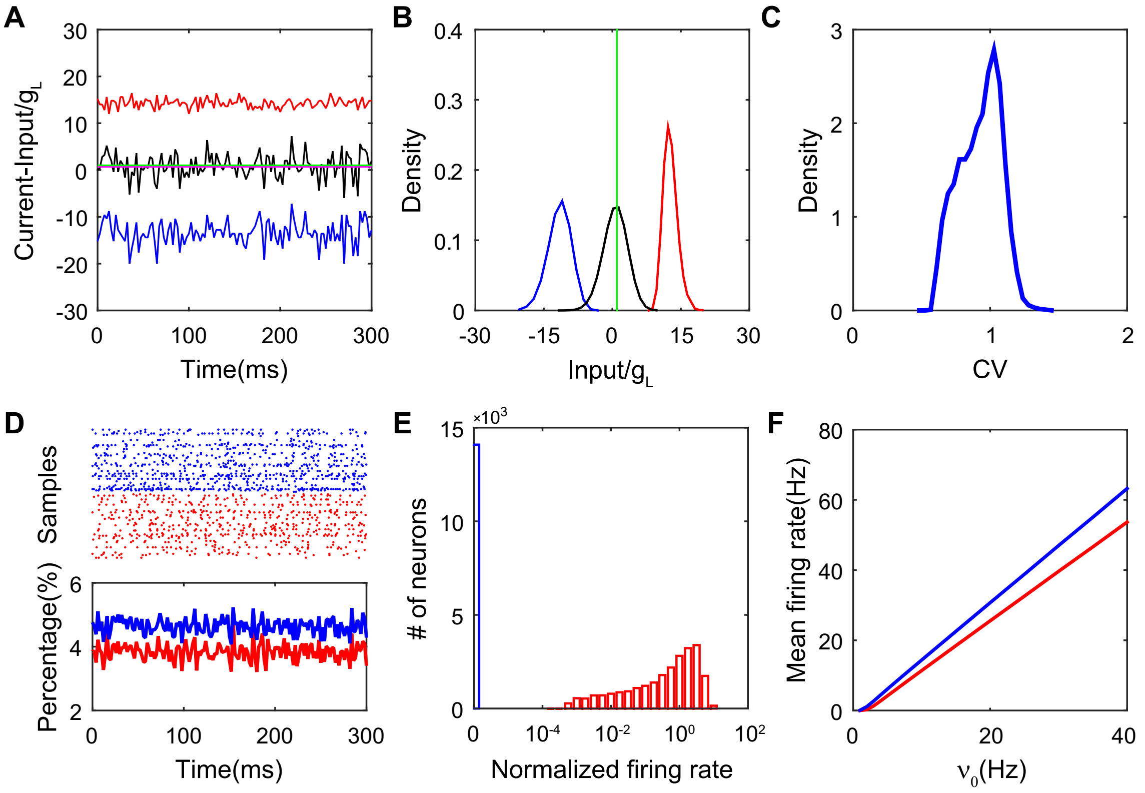

Figure S6. Properties of a balanced SF neuronal network with degree-correlation. (A): The balanced excitatory and inhibitory inputs of a sample neuron (transient dynamics have been removed). The magnitudes of the excitatory (red) and inhibitory (blue) inputs normalized by g_L are much greater than the firing threshold (green), and the total input (black) normalized by g_L has its mean (magenta, the value is 0.67) below the threshold and intermittently crosses it; **(B)**: The probability density functions of the excitatory (red), inhibitory (blue) and total (black) inputs normalized by g_L for the sample neuron in **(A)**. The green line is the threshold; **(C)**: The distribution of CV value of a neuron's ISI over the network. It deviates significantly from zero as the firing activity in the neuronal network is rather irregular; **(D)**: The upper panel is the raster plot of a partial network (100 sample neurons over 300 ms), showing that there is no synchrony; the lower panel shows the percentage of firing neurons in each time window remains nearly constant with small fluctuation over time as the system becomes stationary as time evolves. Here, the transient dynamics have been removed; **(E)**: The log-histogram of neuronal firing rates (normalized by the mean firing rate averaged across the entire network). The blue bar encodes quiescent neurons, and the red bars encode neurons with non-zero firing rates; **(F)**: The mean firing rate of the excitatory and inhibitory populations as a linear function of the external input. Parameters here are the same as those in figure. 1. In panels **(A)**-**(E)**, $\nu_0 = 15$ Hz. The degree correlation of the SF network is $\rho = 0.03$.

S11 Fig

Properties of a balanced SF neuronal network with $\gamma = 1$. The decay exponent of SF network is $\gamma = 1$. Our results in figure. S11 in the Supplemental Data show that there exists a balanced state in this SF neuronal network.

S12 Fig

Properties of a balanced SF neuronal network with $\gamma = 2$. The decay exponent of SF network is $\gamma = 2$. Our results in figure. S12 in the Supplemental Data show that there exists a balanced state in this SF neuronal network.

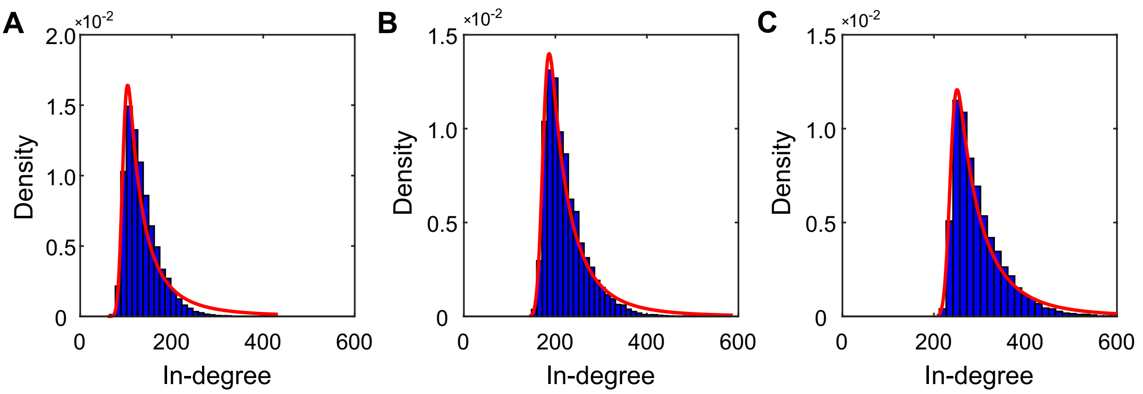

Figure S7. Degree distribution of the active core in SF neuronal networks with different degree correlations. The numerically measured distributions (blue bars) are well captured by the prediction of Eq. (3) (red solid lines), **(A)**: $\rho = -0.3$, **(B)**: $\rho = 0.03$; **(F)**: $\rho = 0.31$.

S13 Fig

Properties of a balanced SF neuronal network with $\gamma = 3$. The decay exponent of SF network is $\gamma = 3$. Our results in figure. S13 in the Supplemental Data show that there exists a balanced state in this SF neuronal network.

S14 Fig

Active cores in SW networks with different rewiring probabilities. We generate the SW network with the following distance-dependent probability: the connected probability between ith and jth neuron is $p_{ij} = qp_0 + (1-q)\Theta(p_0 - d_{ij})$, where $d_{ij} = \min(|i-j|, N-|i-j|)/(N/2)$, N is the total number of neurons in the network, p_0 is the sparsity and q is the rewiring probability Song and Wang (2014). There always exists a balanced active core in neuronal networks with different rewiring probabilities q.

S15 Fig

Firing rate of each firing neuron as a function of its inputs. We choose the rate of the external input to the ith neuron in the αth population ν_α^i from a Gaussian probability distribution with its mean ν_α and standard deviation CV$\cdot\nu_\alpha$ for $\alpha =$ E, I, where CV is the coefficient of variation. We can observe balanced excitatory and inhibitory inputs to the active neurons in all the cases.

S16 Fig

Degree distribution of active core with the heterogeneity in the external input. We choose the rate of the external input to the ith neuron in the αth population ν_α^i from a Gaussian probability distribution with its mean ν_α and standard deviation CV$\cdot\nu_\alpha$ for $\alpha =$ E, I, where CV is the coefficient of variation.

S17 Fig

Heterogeneous input with SF connectivity. We consider a case of using a heterogeneous input into the SF neuronal network. The strength of the external input follows a log-normal distribution. The rate of the external input follows a uniform distribution.

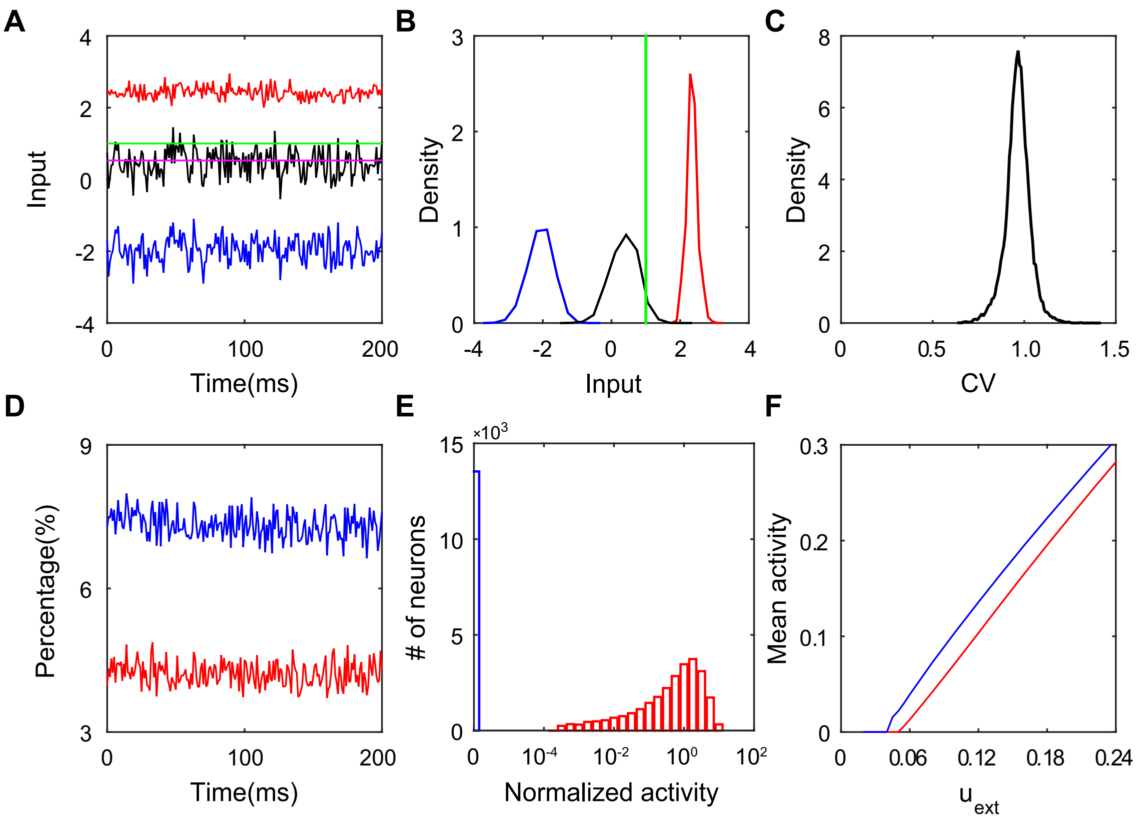

Figure S8. Properties of an SF balanced network with binary neurons. (**A**): The balanced excitatory and inhibitory inputs to a sample neuron (transient dynamics have been removed). The magnitudes of excitatory (red) and inhibitory (blue) inputs are larger than the firing threshold (green), while the mean (magenta, the value is 0.52) of the total input (black) is always smaller than the threshold; (**B**): The probability density functions of the excitatory (red), inhibitory (blue) and total (black) inputs for the sample neuron in panel (**A**). The green line is the threshold; (**C**): The distribution of the CV value. The CV is calculated from the ISIs of each neuron. It is far from zero, signifying the irregularity of neuronal activity; (**D**): The percentage of active neurons in the population at any given time. It signifies a stationary state by staying nearly constant with small fluctuations. The transient dynamics have been removed; (**E**): The log-histogram of neuronal firing rates (normalized by the mean firing rate averaged across the entire network). The blue bar encodes quiescent neurons, and the red bars encode neurons with non-zero firing rates; (**F**): The mean activity of the excitatory population (red solid line) and the inhibitory population (blue solid line) as a linear function of the external input parameter u_{ext}. Parameter values in this simulation are the same as those in Fig. 1. In panels **(A)-(E)**, $u_{\text{ext}} = 0.08$.

REFERENCES

Song, H. F. and Wang, X.-J. (2014). Simple, distance-dependent formulation of the watts-strogatz model for directed and undirected small-world networks. *Physical Review E* 90, 062801

Vreeswijk, C. v. and Sompolinsky, H. (1998). Chaotic balanced state in a model of cortical circuits. *Neural computation* 10, 1321–1371

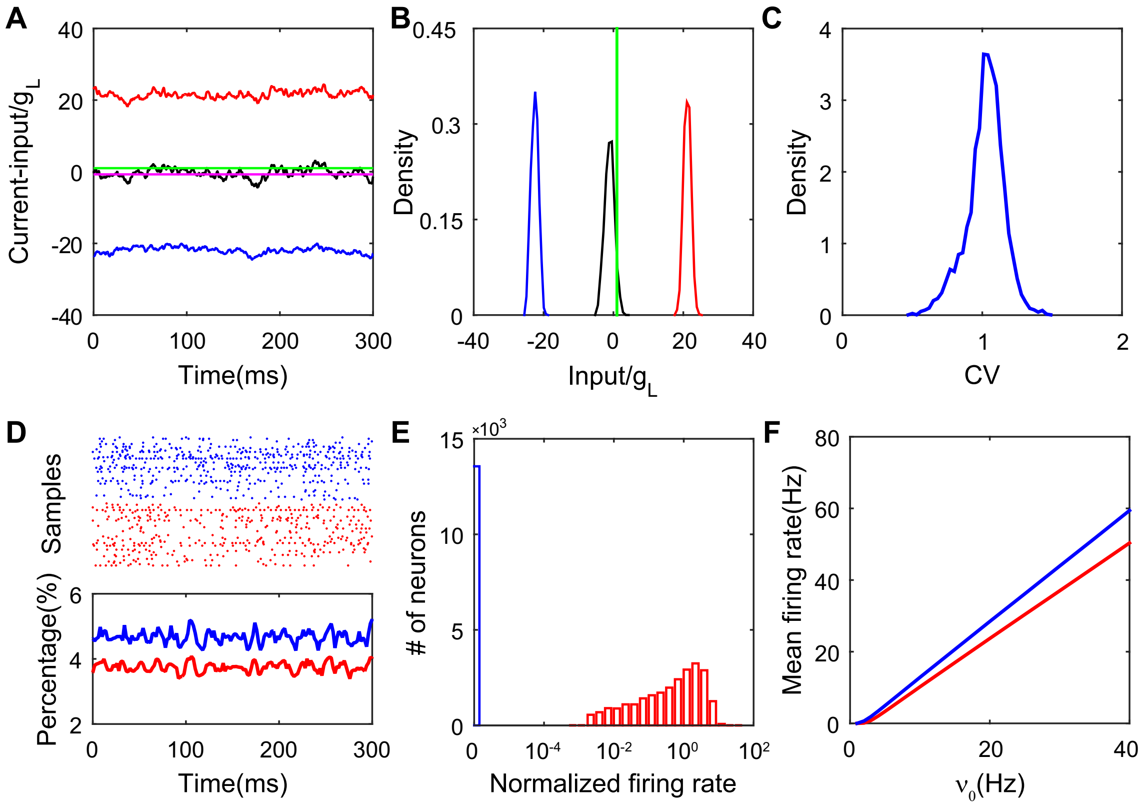

Figure S9. Properties of an SF balanced network with smooth-current-based I&F neurons. (A): The balanced excitatory and inhibitory inputs of a sample neuron (transient dynamics have been removed). The magnitudes of excitatory (red) and inhibitory (blue) inputs normalized by g_L are much larger than the firing threshold (green). Due to the cancellation between excitation and inhibition, the total input normalized by g_L (black) has a small amplitude and occasionally crosses the threshold. The magenta line indicates the mean (the value is -0.69) of the total input; **(B)**: The probability density functions of the excitatory (red), inhibitory (blue) and total (black) inputs (normalized by g_L) for the sample neuron in panel **(A)**. The green line is the threshold; **(C)**: The distribution of the CV value of a neuron's ISI over the network. Since it is far from zero, all spiking neurons in the network fire irregularly; **(D)**: The upper panel is the raster plot of a partial network (100 sample neurons over 300 ms), which exhibits asynchronous neuronal activity; the lower panel shows the percentage of the neurons that spikes over the population in each time window, where the time window is 2.5 ms. The percentage of the spiking neurons in each time window almost keeps constant in time. The transient dynamics have been removed; **(E)**: The log-histogram of neuronal firing rates (normalized by the mean firing rate averaged across the entire network). The blue bar encodes quiescent neurons, and the red bars encode neurons with non-zero firing rates; **(F)**: The mean firing rate of the excitatory and inhibitory populations as a linear function of the external input. Parameters here are the same as those in figure. 1. In panels **(A)-(E)**, $\nu_0 = 15$ Hz.

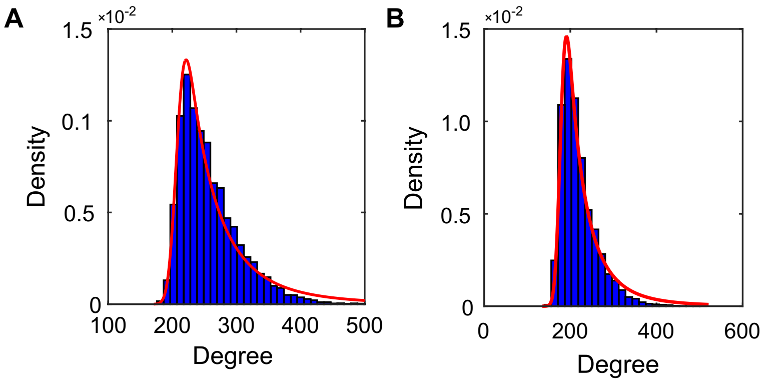

Figure S10. Degree distribution of the active core in the SF network of different type of single neuron models. The measured distribution (blue bars) of the active core for the SF network consisting of binary neurons in **(A)** and current-based I&F neurons with smooth coupling in **(B)** is well captured by the prediction of Eq. (3) (red solid lines). Here the active core is the subnetwork consisting of all the active neurons and the connectivity structure of these active neurons. Data in the panel **(A)** is from the case shown in figure. S8 in the Supplemental Data; data in the panel (B) is from the case shown in figure. S9 in the Supplemental Data.

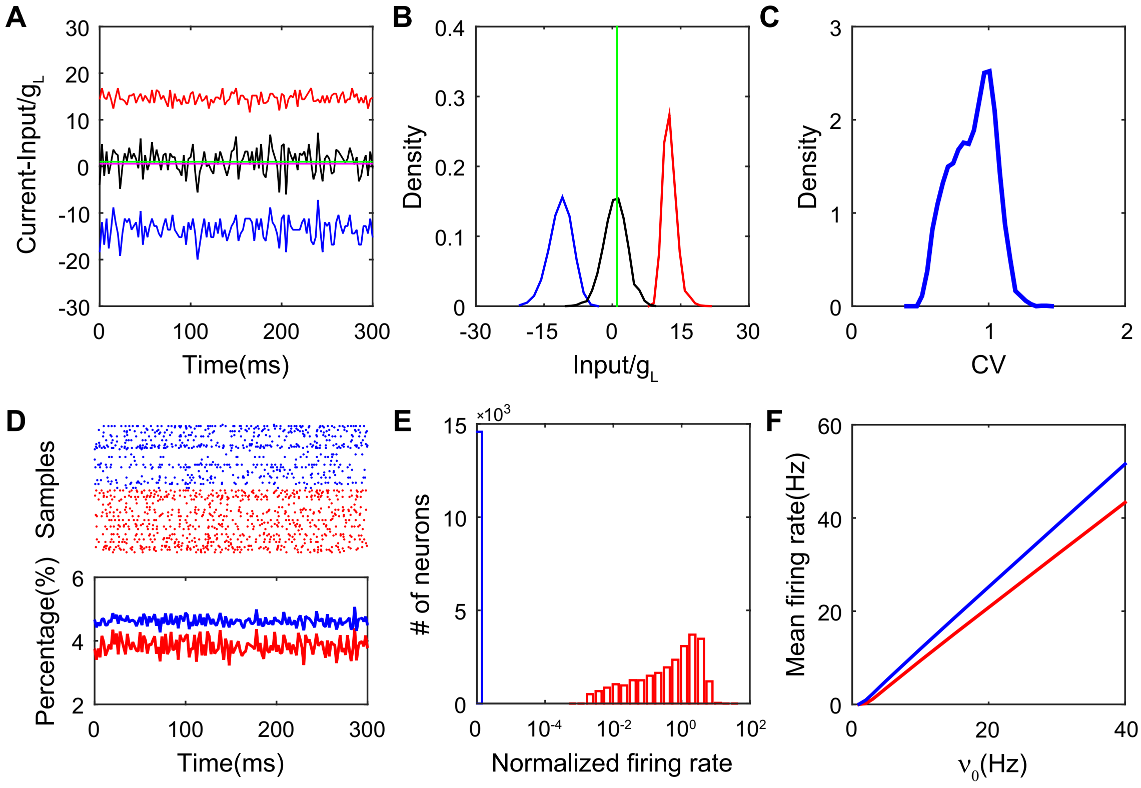

Figure S11. Properties of a balanced SF neuronal network with $\gamma = 1$. (A): The balanced excitatory and inhibitory inputs of a sample neuron (transient dynamics have been removed). The magnitudes of the excitatory (red) and inhibitory (blue) inputs normalized by g_L are much greater than the firing threshold (green), and the total input (black) normalized by g_L has its mean (magenta, the value is 0.02) below the threshold and intermittently crosses it; **(B)**: The probability density functions of the excitatory (red), inhibitory (blue) and total (black) inputs normalized by g_L for the sample neuron in panel **(A)**. The green line is the threshold; **(C)**: The distribution of CV value of a neuron's ISI over the network. It deviates significantly from zero as the firing activity in the neuronal network is rather irregular; **(D)**: The upper panel is the raster plot of a partial network (100 sample neurons over 300 ms), showing that there is no synchrony; the lower panel shows the percentage of firing neurons in each time window remains nearly constant with small fluctuation over time as the system becomes stationary as time evolves. Here, the transient dynamics have been removed; **(E)**: The log-histogram of neuronal firing rates (normalized by the mean firing rate averaged across the entire network). The blue bar encodes quiescent neurons, and the red bars encode neurons with non-zero firing rates; **(F)**: The mean firing rate of the excitatory and inhibitory populations as a linear function of the external input. Parameters here are the same as those in figure. 1. In panels **(A)-(E)**, $\nu_0 = 15$ Hz.

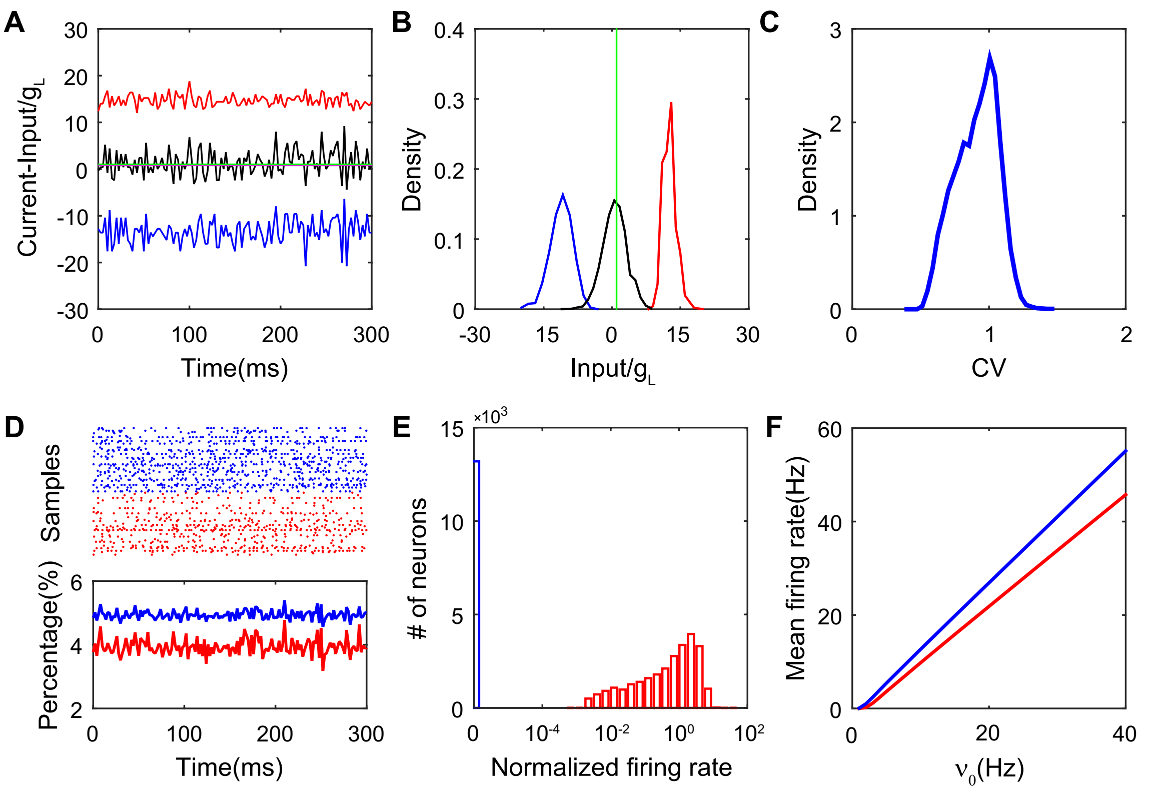

Figure S12. Properties of a balanced SF neuronal network with with $\gamma = 2$. (A): The balanced excitatory and inhibitory inputs of a sample neuron (transient dynamics have been removed). The magnitudes of the excitatory (red) and inhibitory (blue) inputs normalized by g_L are much greater than the firing threshold (green), and the total input (black) normalized by g_L has its mean (magenta, the value is -0.06) below the threshold and intermittently crosses it; **(B)**: The probability density functions of the excitatory (red), inhibitory (blue) and total (black) inputs normalized by g_L for the sample neuron in panel **(A)**. The green line is the threshold; **(C)**: The distribution of CV value of a neuron's ISI over the network. It deviates significantly from zero as the firing activity in the neuronal network is rather irregular; **(D)**: The upper panel is the raster plot of a partial network (100 sample neurons over 300 ms), showing that there is no synchrony; the lower panel shows the percentage of firing neurons in each time window remains nearly constant with small fluctuation over time as the system becomes stationary as time evolves. Here, the transient dynamics have been removed; **(E)**: The log-histogram of neuronal firing rates (normalized by the mean firing rate averaged across the entire network). The blue bar encodes quiescent neurons, and the red bars encode neurons with non-zero firing rates; **(F)**: The mean firing rate of the excitatory and inhibitory populations as a linear function of the external input. Parameters here are the same as those in figure.1. In panels **(A)-(E)**, $\nu_0 = 15$ Hz.

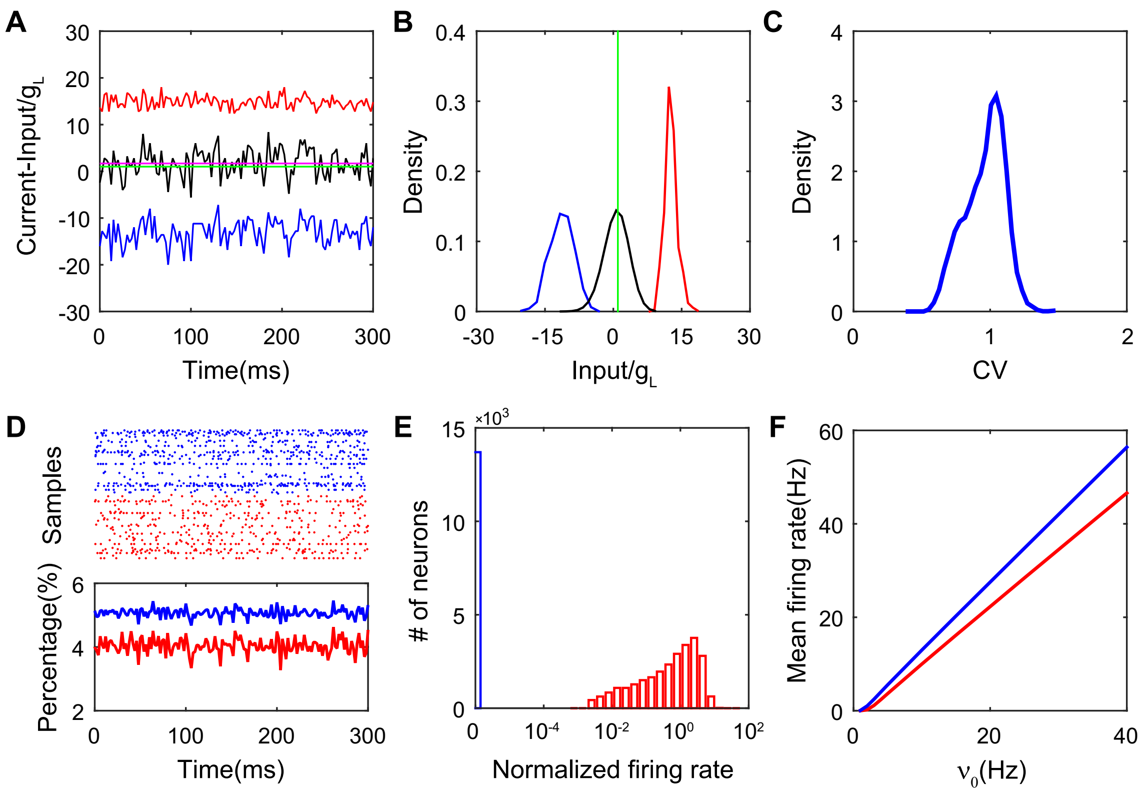

Figure S13. Properties of a balanced SF neuronal network with $\gamma = 3$. (A): The balanced excitatory and inhibitory inputs of a sample neuron (transient dynamics have been removed). The magnitudes of the excitatory (red) and inhibitory (blue) inputs normalized by g_L are much greater than the firing threshold (green), and the total input (black) normalized by g_L has its mean (magenta, the value is 0.01) below the threshold and intermittently crosses it; **(B)**: The probability density functions of the excitatory (red), inhibitory (blue) and total (black) inputs normalized by g_L for the sample neuron in panel **(A)**. The green line is the threshold; **(C)**: The distribution of CV value of a neuron's ISI over the network. It deviates significantly from zero as the firing activity in the neuronal network is rather irregular; **(D)**: The upper panel is the raster plot of a partial network (100 sample neurons over 300 ms), showing that there is no synchrony; the lower panel shows the percentage of firing neurons in each time window remains nearly constant with small fluctuation over time as the system becomes stationary as time evolves. Here, the transient dynamics have been removed; **(E)**: The log-histogram of neuronal firing rates (normalized by the mean firing rate averaged across the entire network). The blue bar encodes quiescent neurons, and the red bars encode neurons with non-zero firing rates; **(F)**: The mean firing rate of the excitatory and inhibitory populations as a linear function of the external input. Parameters here are the same as those in Fig. 1. In panels **(A)**-**(E)**, $\nu_0 = 15$ Hz.

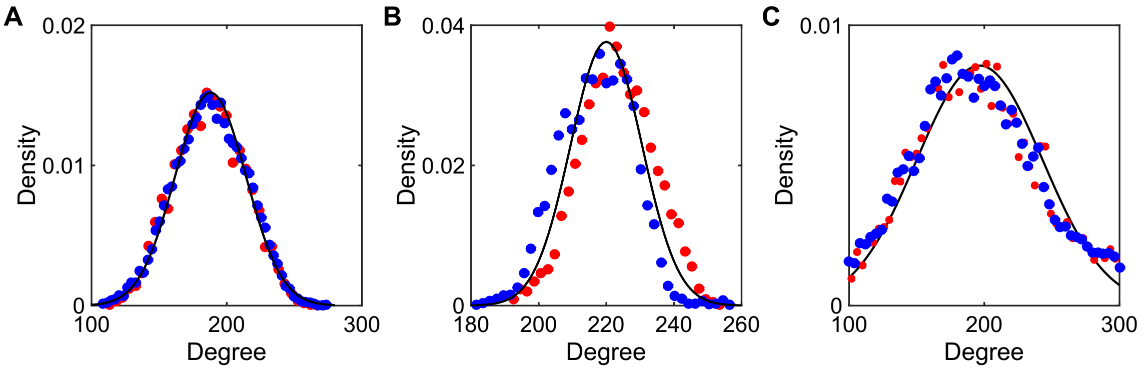

Figure S14. Active cores in SW networks with different rewiring probabilities. (A)-(C): The degree distribution in the active core. Simulation data (dots) compares with the Gaussian distribution (solid line). Red (blue) dots are for connections from presynaptic excitatory (inhibitory) neurons in the active core. The network is generated with the rewiring probability $q = 10^{-1}$ in **(A)**, $q = 10^{-2}$ in **(B)** and $q = 10^{-3}$ in **(C)**

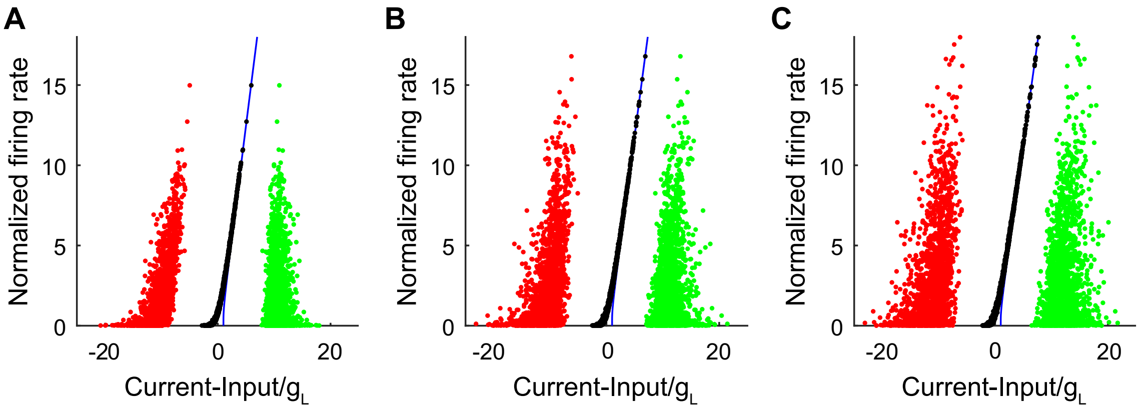

Figure S15. Firing rate of each firing neuron as a function of its inputs. The firing rates of individual neurons vs. the time averaged inhibitory inputs (red dots), excitatory inputs (green dots), net inputs (black dots). The rate of the external input to each neuron is chosen from a Gaussian distribution with coefficient of variation **(A)** CV $= 0.1$; **(B)** CV $= 0.2$; **(C)** CV $= 0.4$. Note that we only plot neurons that fire in simulations and some of the neurons here with small mean input current have small but non-zero firing rates.

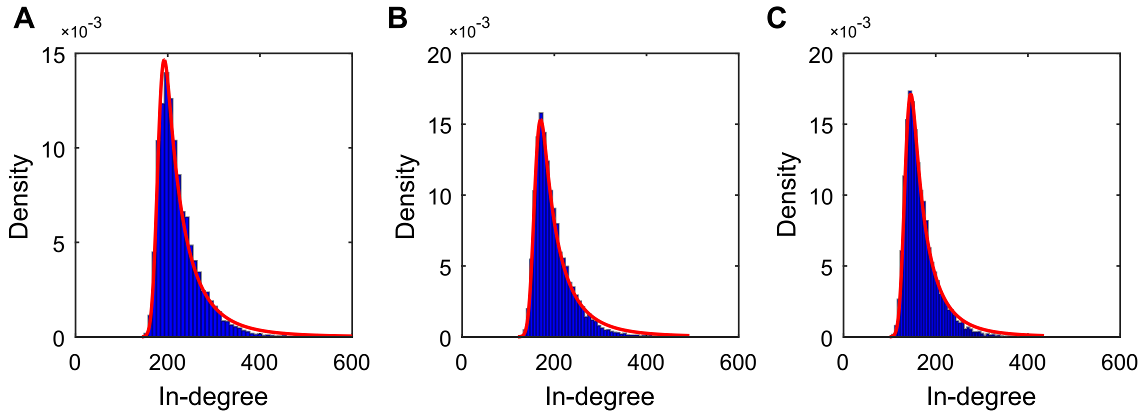

Figure S16. Degree distribution of active core with the heterogeneity in the external input. (A)-(B): The degree distribution in the balanced active core of the SF neuronal network receiving heterogeneous inputs with **(A)** CV $= 0.1$; **(B)** CV $= 0.2$; **(C)** CV $= 0.4$. Measured distribution (blue bars) agrees well with our prediction of Eq. (3) (red line).

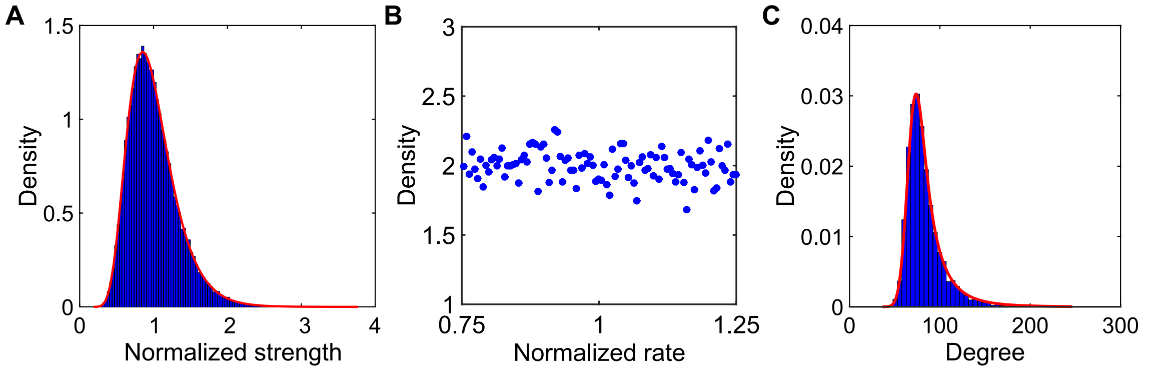

Figure S17. Heterogeneous input (A): The strength distribution of the external input normalized by the mean strength value averaged across the entire network. The simulation data (blue) is fitted by the log-normal distribution(red line); **(B)**: The rate distribution of the external inputs normalized by the mean rate value averaged across the entire network. The rate (blue dot) in our simulations is uniformly distributed; **(C)**: The degree distribution in the balanced active core of the SF neuronal network with heterogeneous inputs. Measured distribution (blue bars) agrees well with our prediction of Eq. (3) (red line). Here we use an SF network with degree correlation $\rho = 0$.
